# Utilisation of the STEAP protein family in a diagnostic setting may provide a more comprehensive prognosis of prostate cancer

**DOI:** 10.1371/journal.pone.0220456

**Published:** 2019-08-08

**Authors:** Stephanie E. A. Burnell, Samantha Spencer-Harty, Suzie Howarth, Owen Bodger, Howard Kynaston, Claire Morgan, Shareen H. Doak

**Affiliations:** 1 Institute of Life Science, Swansea University Medical School, Swansea University, Singleton Park, Swansea, Wales, United Kingdom; 2 Cellular Pathology, Abertawe Bro Morgannwg University Health Board, Singleton Hospital, Sketty Lane, Sketty, Swansea, Wales, United Kingdom; 3 Histopathology, Abertawe Bro Morgannwg University Health Board, Morriston Hospital, Heol Maes Eglwys, Morriston, Swansea, Wales, United Kingdom; 4 Cardiff School of Medicine, Cardiff University, Heath Park, Cardiff, Wales, United Kingdom; University of Minnesota Twin Cities, UNITED STATES

## Abstract

Prostate cancer is the second most common cancer diagnosed in men worldwide; however, few patients are affected by clinically significant disease within their lifetime. Unfortunately, the means to discriminate between patients with indolent disease and those who progress to aggressive prostate cancer is currently unavailable, resulting in over-treatment of patients. We therefore aimed to determine biomarkers of prostate cancer that can be used in the clinic to aid the diagnosis and prognosis. Immunohistochemistry analysis was carried out on prostate cancer specimens with a range of Gleason scores. Samples were stained and analysed for intensity of the Seven Transmembrane Epithelial Antigen of the Prostate (STEAP)-1, -2, -3, -4 and the Divalent Metal Transporter 1 (DMT1) proteins to determine suitable biomarkers for classification of patients likely to develop aggressive prostate cancer. Additionally, these proteins were also analysed to determine whether any would be able to predict future relapse using Kaplan Meier analysis. Data generated demonstrated that the protein expression levels of STEAP2 correlated significantly with Gleason score; furthermore, STEAP4 was a significant predictor of relapse. This data indicates that STEAP2 could be potential prognostic candidate for use in combination with the current prostate cancer detection methods and the presence of STEAP4 could be an indicator of possible relapse.

## Introduction

Prostate cancer (PCa) is the second most common cancer worldwide, however there is currently no successful screening system [1, 2]. Men without symptoms are discouraged from PCa screening by the US Preventive Services Task Force due to the risk of detecting slow growing cancers that will not require treatment within the patients’ lifetime [3]. As slow growing cancers cannot be distinguished from fast growing, aggressive cancers, new prognostic biomarkers are required to improve patient stratification, assist with clinical management of the disease and prevent the overtreatment of PCa patients.

The six-transmembrane epithelial antigen of the prostate (STEAP) family contains four members (STEAP-1, -2, -3 and -4). Proteins containing this 6-transmembrane domain often function as ion channels at cell junctions, which could be a function of the STEAP family, in addition to their suggested metalloreductase function [4]. Due to significant sequence homology with various metalloreductases, it has been suggested that the STEAP family may play a role in iron and copper reduction. Indeed, STEAP2, 3 and 4 expression has been shown to increase iron and copper uptake and promote reduction of iron and copper *in vitro* [5]. Before endocytosed iron can be released from the endosome, into the cell it must be reduced and STEAP, as a ferrireductase, can reduce Fe^3+^ to Fe^2+^ which allows its transport out of the endosome by the divalent metal transporter 1 (DMT1). DMT1 has been shown to be overexpressed in colorectal cancer, consequently dysregulating iron homeostasis, resulting in tumourigenesis [6, 7].

The STEAP protein family has been implicated in many forms of cancer due to overexpression in malignant cells when compared to their non-malignant counterparts. STEAP1 was the first discovered and the smallest member of the STEAP family at only 339 amino acids, as it is missing the N-terminal FNO-like domain [4]. STEAP1 localises to the plasma membrane, it mediates the transfer of small molecules between adjacent cells in culture and is involved in intercellular communication [8, 9]. STEAP1 is expressed in prostate epithelium and at very low levels in a variety of other epithelial tissues including bladder tissue [10]. However, STEAP1 is highly overexpressed in PCa along with 10 other cancers including brain, lung and pancreatic and is associated with poor prognosis and shorter biochemical recurrence survival [10–12]. STEAP2, also known as STAMP1, is primarily expressed in the prostate, with the ovary being the only other significant area of expression [13]. STEAP2 has been shown to shuttle between the Golgi and the plasma membrane before co-localising with early endosomes and it has been speculated to be involved in the processing and/or excretion of prostate specific proteins such as prostate specific antigen (PSA) [14]. The expression of STEAP2 in carcinoma and Benign Prostatic Hyperplasia (BPH) was compared by Porkka *et al*, with results showing that STEAP2 expression was significantly higher in carcinoma than in BPH [13]. STEAP3, also known as STAMP3 and TSAP6 is shown to be expressed at low levels in all tissues but generally decreased in cancer [15]. STEAP3 has been shown contain a p53-response element within the promoter region and to be transcriptionally activated by p53 in response to stress, suggesting a tumour suppressor role of STEAP3, which contrasts the other STEAP proteins [16]. Furthermore, STEAP3 is strongly implicated in iron metabolism and is also reported to be expressed at important iron metabolism sites such as the bone marrow, foetal liver and macrophages, in addition to partially co-localising with transferrin, transferrin receptor and DMT1 all of which are important in the transferrin cycle [17, 18]. This therefore advocates another important role for STEAP3 within iron metabolism. STEAP 4, also known as STAMP2 and TIARP, is primarily expressed in the heart, lung, placenta and prostate with virtually no expression in neuronal tissue [15, 19]. STEAP4 has similar localisation to STEAP2, in addition to localising to the vesicular-tubular structures and co-localising with early endosome antigen 1 (EEA1), suggesting a comparable secretory function to STEAP2 [19]. Silencing of STEAP4 resulted in impairment of insulin-stimulated glucose transport and STEAP4 plays a major role in the protection of cells against metabolic deregulation and inflammatory processes both *in vitro* and *in vivo* [20–22].

This information taken together has led to the hypothesis that the STEAP family, along with DMT1, may be able to identify the extent of disease and therefore aid the prognosis of PCa patients, resulting in the administration of more suitable treatments on a patient by patient basis. The aim of this study was to determine the differences between protein concentration and localisation of each member of the STEAP family, plus DMT1 and observe whether any of these proteins could be utilised in a prognostic fashion in the clinic. We present data here to indicate that STEAP2 may be utilised as a prognostic biomarker, in combination with current methods, due to the significant correlation of STEAP2 expression and Gleason score.

## Materials and methods

Tissue Microarrays (TMAs) were constructed from PCa specimens, from prostatectomy patients, with a range of Gleason scores (n = 209 malignant and n = 47 patient-matched healthy samples) (Wales Cancer Bank (WCB), Cardiff, UK). The Wales Cancer Bank is licensed by the Human Tissue Authority (licence 12107) to store human tissue for research, which has been obtained from patients providing informed consent. Wales Cancer Bank holds ethical approval from Wales Research Ethics Committee 3 to act as a research tissue biobank to both collect and issue biomaterials for cancer related research, where successful peer-reviewed applications have been approved. All methods applied in this report were therefore carried out in accordance with relevant guidelines and regulations in place for Wales Cancer Bank related studies. Prostate samples were bordered and crossed with colorectal tumour tissue to aid orientation. Hematoxylin and eosin (H&E) stained TMA slides were objectively analysed by an independent pathologist to determine that the samples were of a satisfactory standard. Samples with insufficient staining, poor quality of stain, compromised cores (e.g. rolled/partially missing) or lack of tumour cells in the sample were discarded from analysis. Patient information was collected and stored in an anonymised fashion, including Gleason score, PSA levels, age, TNM classification, death and relapse (Table 1).

**Table 1:** Patient Information.

Gleason Score	STEAP1	STEAP2	STEAP3	STEAP4	DMT1	Age Range	PSA Range	Death/ Relapse
Normal	24	28	27	25	27	48–75	2.8–19.6	4
6	24	22	23	23	26	49–71	2.2–19.6	3
3+4 = 7	40	36	37	40	39	49–74	4.5–22.5	2
4+3 = 7	12	12	11	12	12	54–75	2.2–18	3
8	23	24	24	24	27	48–72	3.1–31.7	9
9/10	46	46	48	47	45	43–84	2.2–160	9
Total	169	168	170	171	176	43–84	2.2–160	30

Number of samples available for each biomarker, along with age range, PSA range and number of death and relapse cases for each Gleason score.

The TMA slides were processed using the Benchmark XT automated staining system (ULTRA Ventana, Singleton Hospital, Swansea, UK). Formalin fixed, paraffin embedded sections mounted on FLEX IHC slides were baked in a 60° oven for 1 hour. Barcode labelled slides were placed on the Ventana machine, rinsed with reaction buffer and a liquid cover slip was applied before Ezprep solution was used to dewax the slides at 72°. Following antigen-retrieval, pre-peroxidase inhibitor was applied for 4 minutes at 36°. Antigen retrieval conditions and antibody incubation conditions for each antibody utilised are described in Table 2. OV HQ Universal linker containing secondary antibody was applied (8mins), OV- HPR multimer was applied (8mins). OV DAB and H_2_O_2_ was applied (8mins) before being incubated in copper (4mins) followed by OV AMP multimer (4-8mins). The slides were then counterstained with Heamatoxylin for 8 mins.

**Table 2:** Antibodies and hybridisation conditions utilised in the IHC analysis.

			Retrieval Conditions		Antibody Conditions		
Antibody	Supplier	Positive Control	Buffer	Time (mins)	Dilution	Temperature	Incubation Time (mins)
STEAP1	Abcam	Kidney and prostate	CC1	8	1:200	RT	24
STEAP2	Abcam	Prostate	CC1	32	1:50	36	28
STEAP3	Abcam	Pancreas and prostate	CC1	32	1:80	RT	24
STEAP4	Abcam	Skin and Prostate	CC1	40	1:50	RT	40
DMT1	Abcam	Kidney and Prostate	CC1	40	1:100	36	40

The dilution, temperature and incubation time were optimised for each antibody using the positive controls specified; the final conditions are detailed here. (Cell Conditioning 1 Buffer = CC1 and Room Temperature = RT). Corresponding images of the positive control tissue can be found in S1 Fig.

Samples were scored by two individuals, blind to patient details, based on the intensity (0 = Negative, 1 = Weak, 2 = Moderate and 3 = strong) of the highest stain observed and the percentage (0 = 0%, 1 = 1–25%, 2 = 25–50%, 3 = 51–75% and 4 = 75–100%) of cells stained with this intensity, subcellular localisation was also noted. The final score was then obtained by multiplying the intensity and percentage scores together; defined as: 0 = Negative, 1–3 = Weak, 4–6 = Moderate and 8–12 = Strong [23, 24].

### Statistical analysis

Statistical analysis was performed using SPSS software version 22. Pearson test to assess the correlation between biomarker, Gleason, PSA and age. Differences between means (two samples) were analysed using the independent samples t-test and >two samples were analysed a one-way ANOVA. A p-value of <0.05 was significant. Kaplan Meier plots were carried out to determine if there was a significant difference in the time taken to relapse. Kaplan Meier analysis was performed using SPSS and the long rank test was utilised to analyse this data. Due to requirement for normally distributed data PSA values were log transformed to produce a normal distribution.

## Results

The protein expression of the STEAP family, along with DMT1 were analysed in PCa samples with varying Gleason scores using IHC. In addition to the Gleason score of the patient samples, PSA level, age and relapse information was provided as stated in Table 1. Comparisons between each potential biomarker and the patient information were carried out to observe the relationship between current prognostic methods and identification of aggressive PCa. This evaluation was aimed at determining whether additional prognostic information required for successful clinical management of PCa patients could be fulfilled by evaluation the STEAP protein family.

### STEAP2 protein expression significantly correlates with Gleason score

**Fig 1:**
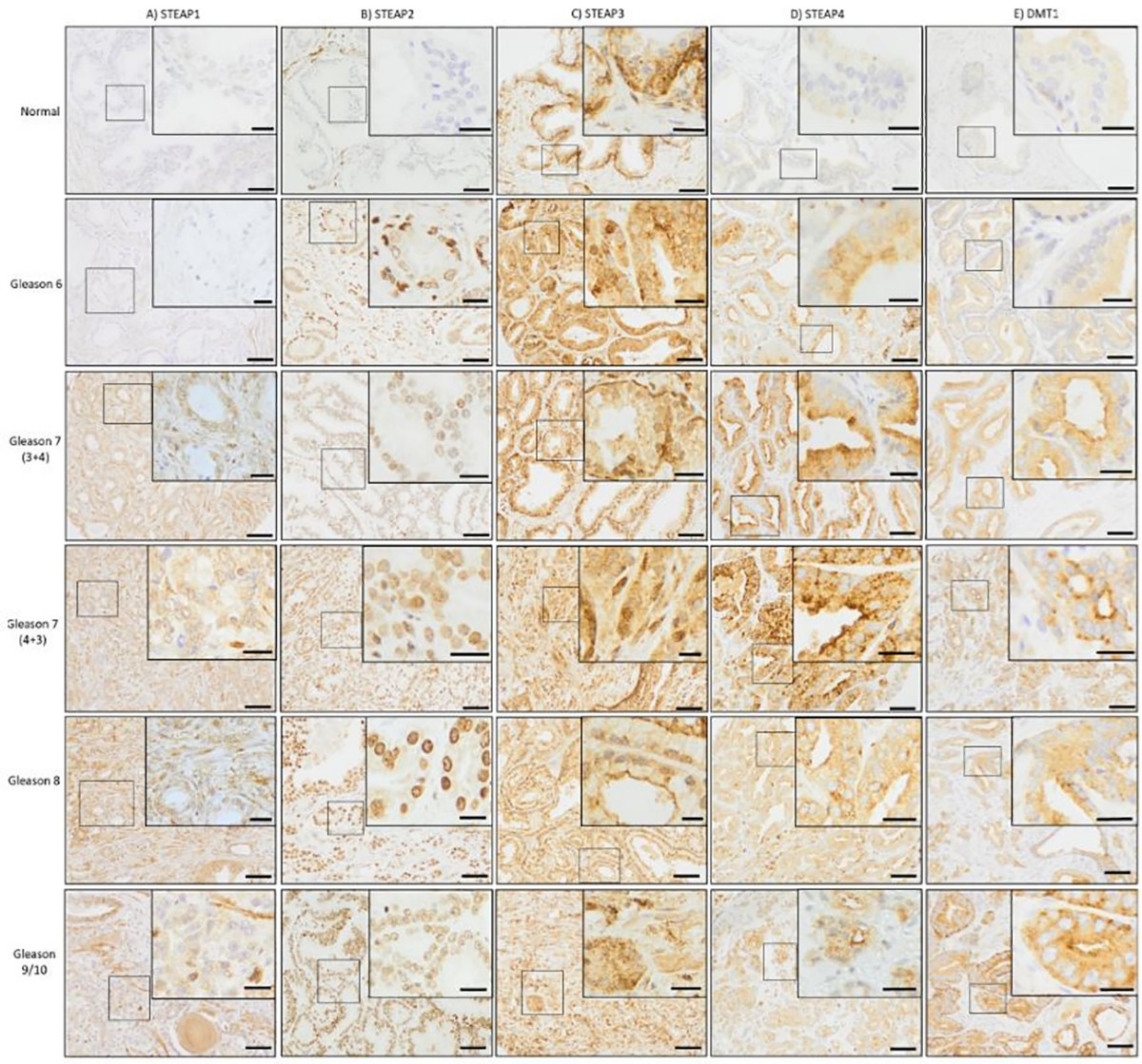
Alterations in biomarker protein expression compared against Gleason score. Panel shows the differences in protein expression of the 4 STEAP family members plus DMT1 with regards to increasing Gleason score. (A) STEAP1 expression increases from the normal sample to the cancer samples and increases from Gleason 6 to Gleason 7. From Gleason 7, the protein expression appears to decrease in a step-wise manner towards Gleason 9/10. (B) STEAP2 expression increases from the normal samples to the cancer samples, and then continues to increase in a step-wise manner towards Gleason 9/10. (C) STEAP3 protein expression is consistently high in all samples observed. (D) STEAP4 protein expression increases from the normal to cancer samples and remains high. The highest protein expression is observed in the Gleason 7 samples. (E) DMT1 protein expression increases from the normal to cancer samples, then remains consistently high throughout. Scale bars represent 50 μm (main image) and 20 μm (insert).

**Fig 2:**
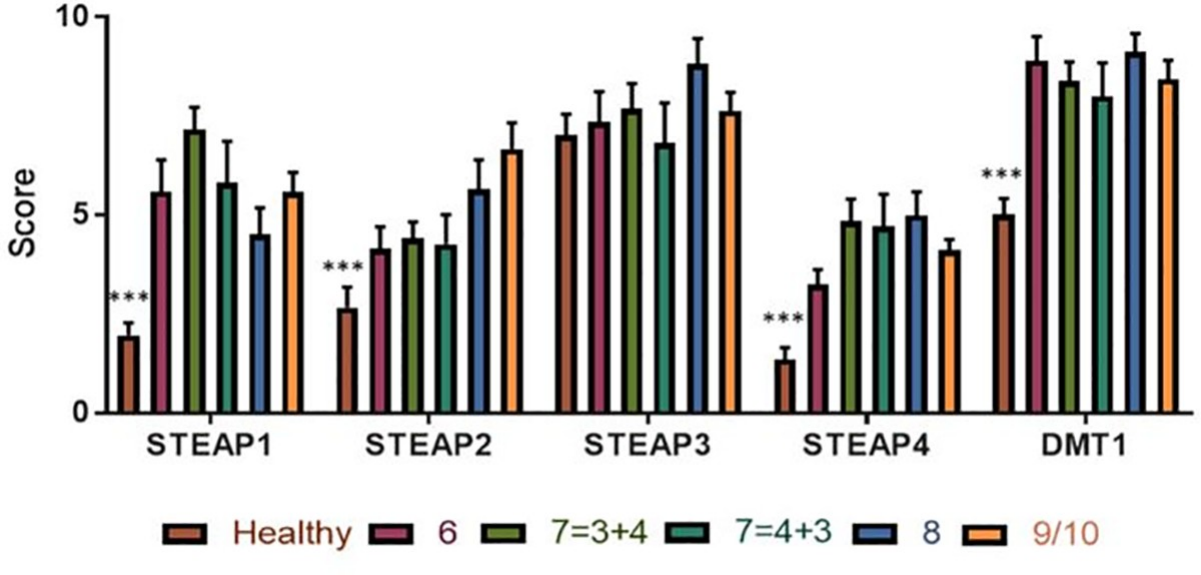
Correlation between biomarker and Gleason score. Bars show Healthy patients against Gleason score breakdown. STEAP1, 2, 4 and DMT1 protein expression was significantly increased in PCa samples compared to healthy samples. Significant positive correlation (r = 0.308, p<0.001) was observed for STEAP2 against Gleason scores. Significance between healthy vs cancer samples (independent samples, two-tailed t-test) denoted as *<0.05, **<0.01, ***<0.001.

The protein concentration and localisation of each biomarker was observed and compared to Gleason score (Figs 1 and 2). STEAP1 expression was significantly higher in the PCa specimens relative to the normal prostate specimens (p<0.001) and was observed to localise to the cytoplasm of all cells. The STEAP1 stain intensity in the normal samples was weak, the staining increased slightly in the Gleason 6 samples and then dramatically in the Gleason 7 (3+4 and 4+3) onwards.

STEAP2 expression was significantly higher level in the PCa specimens relative to the normal prostate specimens (p<0.001) with a positive correlation between STEAP2 and Gleason score (r = 0.308, p<0.001). STEAP2 expression was very low in the normal prostate specimens with only minimal staining present in the cytoplasm. In contrast to this, STEAP2 was observed to localise to the nucleus in the PCa specimens. Stain intensity was increased dramatically in the Gleason 6 samples. From Gleason 7 (3+4 and 4+3) the intensity of STEAP2 stain increased in a step wise fashion to Gleason 9/10.

STEAP3 expression was high in all samples and present at the cell membranes and the cytoplasm, with no difference between healthy and cancer samples and no correlation with regards to Gleason score.

STEAP4 expression was very low in the normal prostate specimens with only minimal protein expression present in the cytoplasm. In contrast, STEAP4 localises to the lumen, with heavy cytoplasmic staining in all cells of the PCa specimens. STEAP4 expression was significantly higher in the PCa specimens relative to the normal prostate specimens (p<0.001), although there was no correlation between STEAP4 and Gleason scores.

DMT1 expression was very low in the normal prostate specimens, present only in the cytoplasm. In contrast, DMT1 protein expression is dramatically increased and is observed to localise to the cytoplasm of all PCa specimens. DMT1 expression was significantly higher level in the PCa specimens when compared to the normal prostate specimens (p<0.001). No correlation was observed between DMT1 and Gleason score, according to Pearson correlation tests.

### No correlation between LogPSA/Age and biomarker score

**Fig 3:**
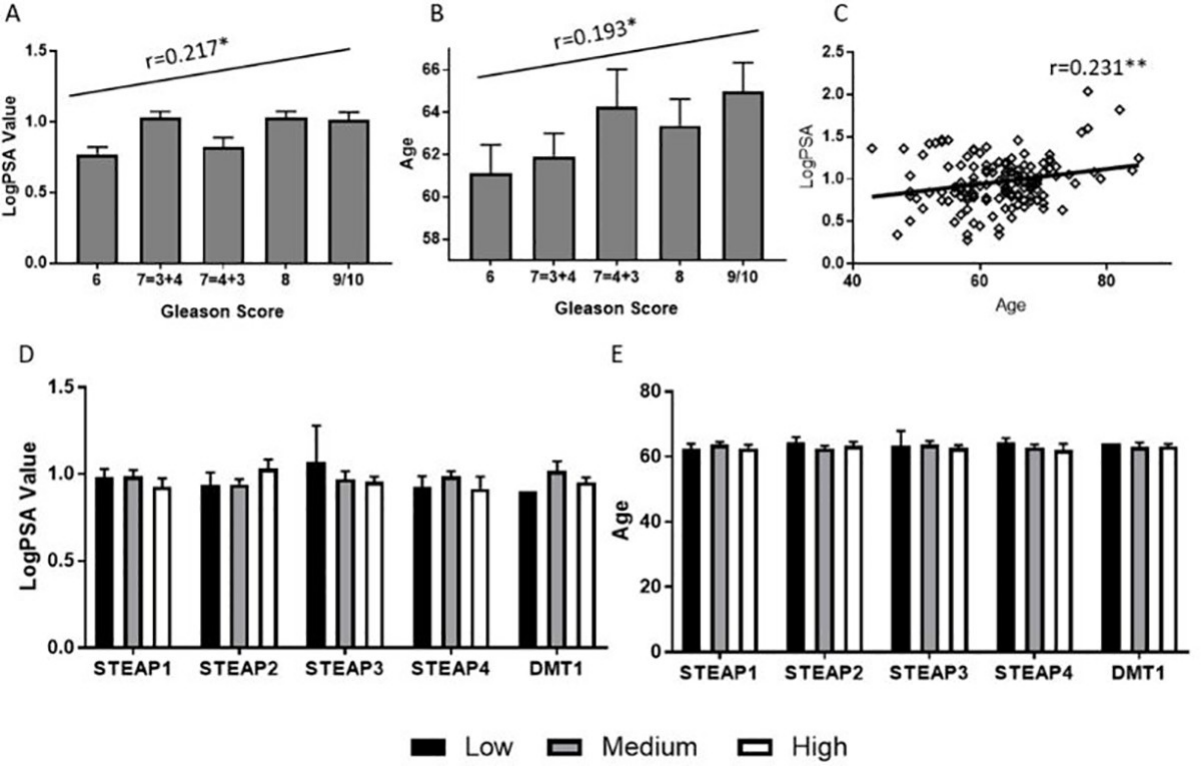
Correlations of PSA Value, Age, Gleason score and biomarker score. Both (A) LogPSA value and (B) Age correlate positively with Gleason score (r = 0.217, p = 0.011 and 0.193, p = 0.023 respectively, Pearson test). (C) A positive correlation was also observed between LogPSA and age (r = 0.231, p = 0.006, Pearson test). No correlation was observed between STEAP1, 2, 3, 4 or DMT1 with (D) PSA value or (E) Age. Significance denoted as *<0.05, **<0.01, ***<0.001.

The two common factors in the progression of PCa are PSA value and age. As Gleason score increases, we have observed that the PSA value and the age of the patient also increase (r = 0.217, p = 0.011 and 0.193, p = 0.023 respectively) (Fig 3A and 3B), which indicates that these two factors can predict the Gleason score of the patient. However, as shown in Fig 3C, PSA value and age are also significantly positively correlated (0.231, p = 0.006), indicating that there may be other factors influencing the increase of PSA in the body. For a biomarker to be capable of predicting prognostic outcome, it is important that it is not affected by any other confounding factor. In the present study, biomarker score was split into three groups: Low (0–2), Medium (3–6) and High (8–12) and we found no correlation with either PSA value or age for any of the biomarkers analysed (Fig 3D and 3E).

### Chance of relapse was predicted by Gleason score, PSA value and age, while time taken to relapse was predicted by STEAP4 expression

**Fig 4:**
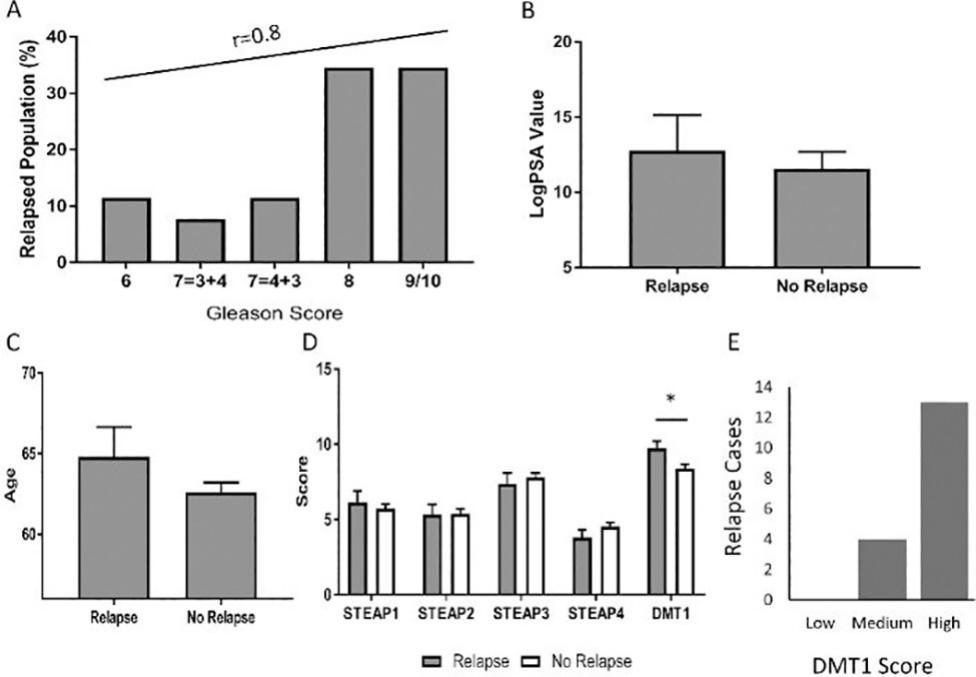
Chance of relapse was increased with increasing Gleason Score, PSA level and age. (A) Gleason, (B) PSA level and (C) Age were plotted against the death and relapse of the patient. There was a positive correlation between increasing Gleason score and relapsing patients. (D) Relapse was significantly more likely with a higher DMT1 score (p = 0.037, independent samples, two-tailed t-test) and (E) An increased number of relapse cases occur with a higher DMT1 score. (High group n = 13, medium group n = 4, low group n = 0). Significance denoted as *<0.05, **<0.01 and ***<0.001.

It is important for a prognostic biomarker to be able to determine the likely progression of the disease. Fig 4A shows a positive correlation between increasing Gleason score and relapsing population (r = 0.8). Fig 4B and 4C show that patients with increasing PSA levels and age were more likely to suffer from death and relapse, however, this was not significant. Fig 4D and 4E indicate that relapse was more likely to occur in patients with increased DMT1 expression (p = 0.037, independent samples, two-tailed t-test). A binary logistic regression was carried out to determine the true prognostic value of DMT1, this analysis showed the odds of relapse increased by 20.8% per unit increase of DMT1 (p = 0.037). This increases to 22.7% per unit of DMT1 score (p = 0.028) when controlling for Gleason, PSA and Age. This therefore indicates that the prognostic value of DMT1 stands true when controlling for the other variable, namely Gleason score, PSA value and age. Fig 4E illustrates the increase of relapse cases with respect to DMT1 score, where score was split into three groups: Low (0–2), Medium (3–6) and High (8–12). No Significant difference was observed in any STEAP biomarker expression with regards to relapse (Fig 4D).

**Fig 5:**
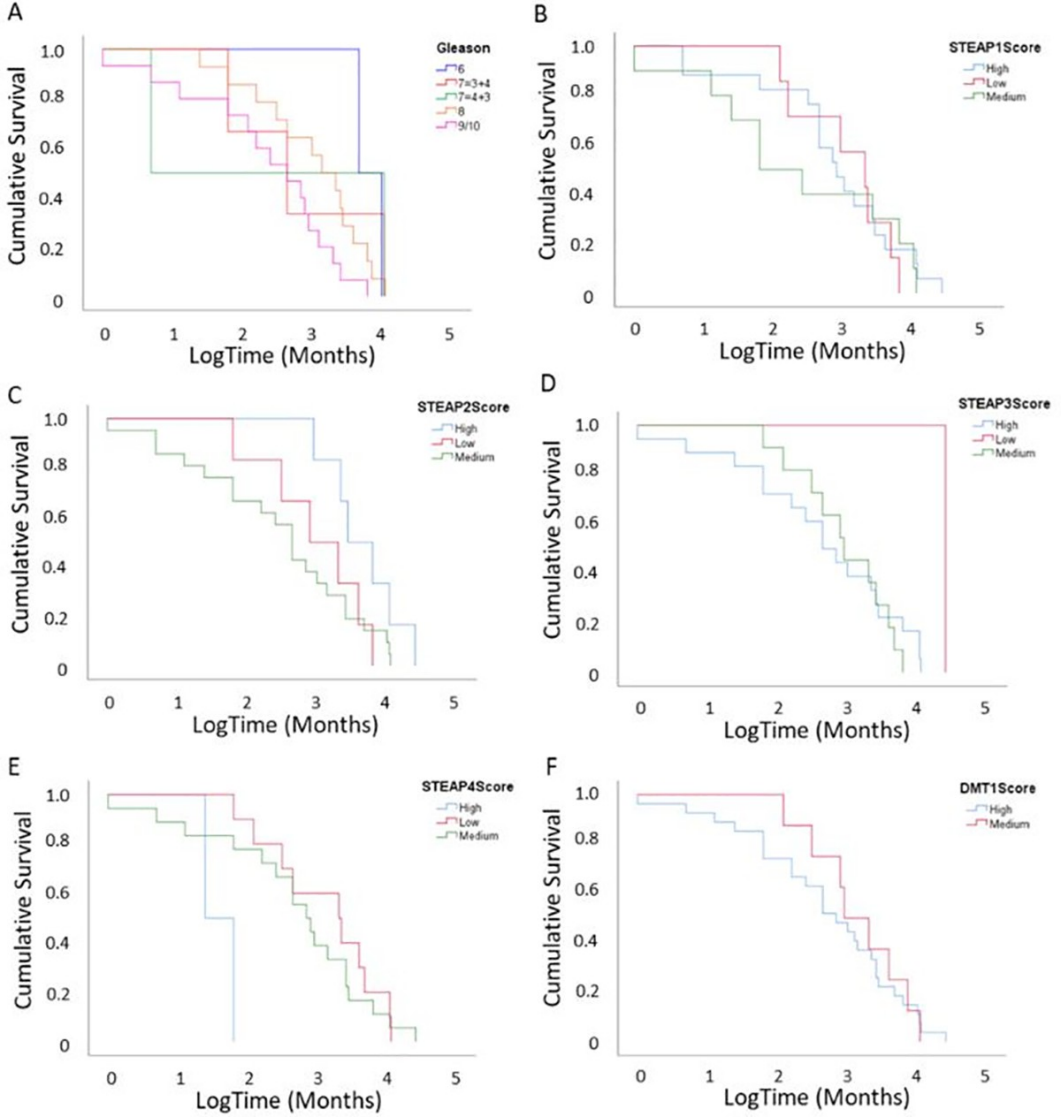
Kaplan Meier curve as a function of Gleason score, STEAP1, 2, 3, 4 and DMT1 Score. (A) Patients with higher Gleason scores are more likely to progress to relapse than those with lower Gleason scores, although there is no significant difference in time taken to progress to relapse between all Gleason scores. (B) Patients with higher STEAP1 score are more likely to progress to relapse than those with medium and low scores, however, there was no significant difference. (High group n = 17, medium group n = 10 and low group n = 7) (C) Patients with higher STEAP2 score are more likely to progress to relapse than those with medium and low scores, however, there was no significant difference. (High group n = 6, medium group n = 21 and low group n = 6) (D) Patients with lower STEAP3 score are less likely to progress to relapse than those with medium and high scores, however this was not significant. (High group n = 18, medium group n = 11 and low group n = 1) (E) Patients with higher STEAP4 score were significantly more likely to progress to relapse earlier than those with medium or low scores (p = 0.025, long rank test). (High group n = 2, medium group n = 18 and low group n = 10) (F) There was no difference in time taken to relapse between high and medium DMT1 scores. (High group n = 27 and medium group n = 8).

Kaplan Meier graphs were used to evaluate the relationship between the test biomarker expression and time to relapse, we observed no significant association for Gleason, STEAP1, STEAP2, STEAP3 and DMT1 scores (Fig 5A, 5B, 5C, 5D and 5F). In contrast, STEAP4 was the only biomarker showing significant association with relapse suggesting that patients with a high STEAP4 protein expression relapsed more quickly than those with medium or low STEAP4 protein expressions (Fig 5E).

## Discussion

Currently, Gleason scoring is the gold standard for predicting PCa outcome; however, the Gleason score is subjective, being dependent on the pathologist’s judgement. It is therefore important that additional biomarkers are identified that are more predictive of patients likely to develop advanced disease. In the present study, all members of the STEAP protein family were analysed to determine their suitability to improve the prognostic prediction of PCa. In addition to the analysis of STEAP1-4, DMT1, was also analysed. There are significant positive correlations between each of Gleason, PSA and age indicating they may be closely related. These observations are supported by the literature, where correlations between these parameters have previously been established [25–27]. In particular, the increase of PSA level with age is widely documented and has resulted in the introduction of age-specific PSA reference ranges in an attempt to increase the accuracy of PSA testing [28]. Differing PSA levels have also been linked to race, with some studies suggesting not only age-specific, but race-specific PSA ranges for the detection of PCa [29]. This information presents a poor case for PSA as a diagnostic tool as there are multiple factors not related to tumourigenesis that influence PSA level.

In the present IHC study, each biomarker protein expression score was correlated to Gleason score and these results indicated a significant positive correlation between STEAP2 and Gleason score. This finding contradicts one previous study that reported no significant association between STEAP2 and Gleason score [30]. However, the sample size used by the Wang *et al* study (n = 67) was approximately half the samples used the present study and so possibly lacking in power.

Since Gleason, PSA and age are all closely interlinked and elevated STEAP2 protein expression levels are significantly correlated with increasing Gleason score, it was important to determine whether the protein expression of STEAP-1-, -2, -3, -4 and DMT1 were correlated to PSA level and age. A biomarker capable of predicting prognostic outcome that was not affected by other factors would be beneficial. No correlation was found between any STEAP biomarker and PSA and age respectively supporting the suitability of STEAP2 as a prognostic test for aggressive PCa.

Although, STEAP4 was the only biomarker to be significantly associated with patient relapse it was notable that the number of relapsing patients was very small (n = 36 out of 185 patients). These results therefore would benefit from being validated with a larger patient cohort.

In conclusion, we suggest that STEAP1, 2, 4 and DMT1 are suitable candidates to distinguish patients with cancer from those that have no tumour present. When the correlation with Gleason score was taken into consideration, STEAP2 presents as a potential prognostic biomarker, although on its own, it is not a solid indicator of the extent of disease. We therefore propose STEAP2 to be used in combination with existing PCa screening methods, (e.g. PSA testing, DRE examination and biopsy analysis). Although studies with larger cohorts of patients are required, this biomarker is particularly promising as a predictor of prognostic outcome due to the lack of association with confounders such as PSA levels and age.

## Supporting information

S1 Fig Immunohistochemistry positive control tissue staining for STEAP1-4 and DMT1. Kidney and prostate tissues were used as positive controls for STEAP1 and DMT1 IHC, prostate tissue was used as a positive control for STEAP2 IHC, pancreas and prostate tissues were used as positive controls for STEAP3 IHC and skin and prostate tissues were used as positive controls for STEAP4 IHC. Prostate tissue was from adenocarcinoma, all other tissue was healthy. Scale bars represent 50 μm (main image) and 20 μm (insert).
(TIF)

S1 File Raw data used to draw conclusions in this manuscript. Tables summarising the raw data used for Table A) relationship between Gleason score and biomarker score, Table B) difference in biomarker score between tumour tissue and corresponding healthy tissue, Table C) correlations between biomarker score, PSA and age, Table D) effect of biomarker score on the chance of relapse, Table E) prognostic value of the biomarkers and Table F) Kaplan Meier plots.
(DOCX)
