# Supplementary figures and images for: Utilisation of the STEAP protein family in a diagnostic setting may provide a more comprehensive prognosis of prostate cancer
Source: PLoS One. 2019 Aug 8;14(8):e0220456. doi: 10.1371/journal.pone.0220456 (PMC6687176; doi:10.1371/journal.pone.0220456)

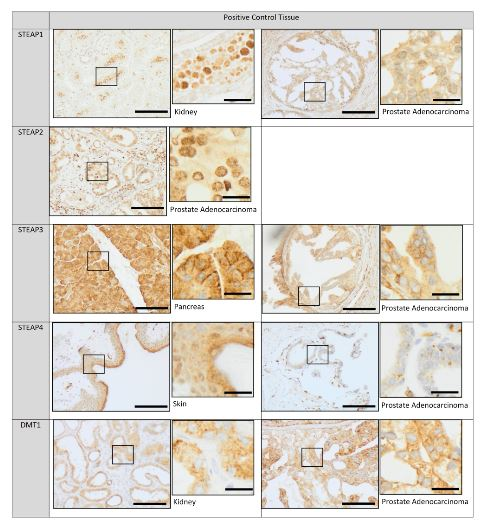

Supplement: S1 Fig — Kidney and prostate tissues were used as positive controls for STEAP1 and DMT1 IHC, prostate tissue was used as a positive control for STEAP2 IHC, pancreas and prostate tissues were used as positive controls for STEAP3 IHC and skin and prostate tissues were used as positive controls for STEAP4 IHC. Prostate tissue was from adenocarcinoma, all other tissue was healthy. Scale bars represent 50 μm (main image) and 20 μm (insert). (TIF) [file pone.0220456.s001.tif]
