# Supplementary material for: Utilisation of the STEAP protein family in a diagnostic setting may provide a more comprehensive prognosis of prostate cancer
Source: PLoS One. 2019 Aug 8;14(8):e0220456. doi: 10.1371/journal.pone.0220456 (PMC6687176; doi:10.1371/journal.pone.0220456)
Supplement: S1 File — Tables summarising the raw data used for Table A) relationship between Gleason score and biomarker score, Table B) difference in biomarker score between tumour tissue and corresponding healthy tissue, Table C) correlations between biomarker score, PSA and age, Table D) effect of biomarker score on the chance of relapse, Table E) prognostic value of the biomarkers and Table F) Kaplan Meier plots. (DOCX) [file pone.0220456.s002.docx]

**Supporting Information File**

1. Raw data used to determine the relationship between Gleason score and biomarker score (Figure 2).

| Patient Number | Gleason Score | STEAP1 Score | STEAP2 Score | STEAP3 Score | STEAP4 Score | DMT1 Score |
| --- | --- | --- | --- | --- | --- | --- |
| 001 | 7=3+4 | 4 | 4 | 8 | 0 | 6 |
| 002 | 9/10 | 4 | 0 | 4 | 4 | 4 |
| 003 | 7=4+3 | 4 | 4 | 8 | 12 | 12 |
| 004 | 6 | 12 | 4 | 8 | 4 | 9 |
| 005 | 8 | 0 | 6 | 8 | 4 | 8 |
| 006 | 8 | 6 | 2 | 8 | 2 | 12 |
| 007 | 9/10 | 8 | 4 | 8 | 0 | 12 |
| 008 | 6 | 4 | 9 | 12 | 4 | 6 |
| 009 | 7=4+3 | 4 | 2 | 2 | 6 | 8 |
| 010 | 9/10 | 2 | 2 | 4 | 4 | 9 |
| 011 | 9/10 | 6 | 3 | 6 | 4 | 12 |
| 012 | 6 | 0 | 4 | 4 | 3 | 4 |
| 013 | 7=4+3 | 3 | 9 | 12 | 4 | 12 |
| 014 | 9/10 | 4 | 8 | 12 | 4 | 9 |
| 015 | 6 | 3 | 6 | 6 | 4 | 12 |
| 016 | 7=3+4 | 4 | 4 | 2 | 4 | 9 |
| 017 | 9/10 | 6 | 2 | 8 | 4 | 12 |
| 018 | 7=3+4 | 6 | 6 | 6 | 4 | 12 |
| 019 | 8 | 6 | 2 | 8 | 4 | 9 |
| 020 | 9/10 | 2 | 4 | 8 | 4 | 8 |
| 021 | 8 | 4 | 2 | 12 | 2 | 12 |
| 022 | 9/10 | 0 | 12 | 4 | 3 | 6 |
| 023 | 6 | 4 | 4 | 8 | 4 | 9 |
| 024 | 7=3+4 | 4 | 1 | 6 | 8 | 8 |
| 025 | 8 | 3 | 6 | 8 | 4 | 9 |
| 026 | 8 | 9 | 4 | 12 | 6 | 9 |
| 027 | 9/10 | 0 | 6 | 8 | 4 | 12 |
| 028 | 7=3+4 | 3 | 4 | 4 | 4 | 6 |
| 029 | 7=3+4 | 6 | 4 | 12 | 3 | 12 |
| 030 | 8 | 0 | 0 | 3 | 4 | 8 |
| 031 | 6 | 8 | 4 | 6 | 4 | 8 |
| 032 | 8 | 12 | 1 | 4 | 2 | 6 |
| 033 | 7=3+4 | 12 | 2 | 12 | 4 | 6 |
| 034 | 9/10 | 4 | 3 | 8 | 4 | 12 |
| 035 | 8 | 4 | 3 | 8 | 6 | 9 |
| 036 | 6 | 0 | 4 | 4 | 0 | 8 |
| 037 | 7=3+4 | 8 | 4 | 6 | 4 | 8 |
| 038 | 6 | 4 | 6 | 9 | 4 | 9 |
| 039 | 7=3+4 | 6 | 4 | 9 | 2 | 9 |
| 040 | 7=4+3 | 4 | 6 | 8 | 4 | 9 |
| 041 | 9/10 | 8 | 0 | 4 | 4 | 6 |
| 042 | 6 | 9 | 0 | 4 | 4 | 8 |
| 043 | 7=4+3 | 2 | 4 | 4 | 6 | 2 |
| 044 | 9/10 | 8 | 4 | 8 | 4 | 12 |
| 045 | 6 | 3 | 2 | 6 | 3 | 6 |
| 046 | 7=4+3 | 8 | 4 | 12 | 4 | 8 |
| 047 | 7=4+3 | 12 | 4 | 8 | 4 | 8 |
| 048 | 9/10 | 2 | 0 | 4 | 2 | 4 |
| 049 | 6 | 4 | 4 | 12 | 6 | 12 |
| 050 | 7=3+4 | 8 | 2 | 6 | 4 | 9 |
| 051 | 7=3+4 | 4 | 8 | 8 | 4 | 9 |
| 052 | 7=3+4 | 12 | 4 | 12 | 8 | 9 |
| 053 | 6 | 6 | 4 | 12 | 4 | 12 |
| 054 | 7=4+3 | 6 | 3 | 4 | 3 | 9 |
| 055 | 7=3+4 | 12 | 4 | 6 | 4 | 8 |
| 056 | 6 | 6 | 3 | 4 | 4 | 9 |
| 057 | 7=3+4 | 12 | 4 | 12 | 8 | 9 |
| 058 | 7=3+4 | 12 | 4 | 8 | 4 | 4 |
| 059 | 6 | 8 | 4 | 9 | 3 | 12 |
| 060 | 7=4+3 | 6 | 2 | 6 | 2 | 6 |
| 061 | 7=3+4 | 4 | 1 | 8 | 12 | 8 |
| 062 | 6 | 4 | 4 | 12 | 2 | 12 |
| 063 | 7=3+4 | 8 | 6 | 12 | 3 | 12 |
| 064 | 8 | 0 | 4 | 4 | 6 | 12 |
| 065 | 7=3+4 | 4 | 6 | 12 | 12 | 6 |
| 066 | 7=3+4 | 6 | 9 | 8 | 8 | 9 |
| 067 | 7=3+4 | 0 | 6 | 4 | 4 | 8 |
| 068 | 7=3+4 | 8 | 2 | 8 | 4 | 9 |
| 069 | 6 | 3 | 9 | 9 | 4 | 12 |
| 070 | 7=3+4 | 8 | 2 | 3 | 6 | 6 |
| 071 | 7=3+4 | 9 | 4 | 9 | 4 | 6 |
| 072 | 7=3+4 | 6 | 3 | 4 | 6 | 12 |
| 073 | 7=3+4 | 6 | 4 | 6 | 2 | 12 |
| 074 | 7=3+4 | 12 | 6 | 12 | 8 | 12 |
| 075 | 8 | 4 | 4 | 12 | 12 | 8 |
| 076 | 8 | 4 | 12 | 4 | 0 | 6 |
| 077 | 8 | 4 | 12 | 9 | 6 | 6 |
| 078 | 9/10 | 4 | 9 | 9 | 9 | 12 |
| 079 | 9/10 | 4 | 6 | 6 | 6 | 8 |
| 080 | 8 | 4 | 4 | 12 | 9 | 12 |
| 081 | 9/10 | 4 | 8 | 12 | 4 | 6 |
| 082 | 8 | 4 | 9 | 6 | 4 | 8 |
| 083 | 9/10 | 0 | 9 | 2 | 6 | 8 |
| 084 | 9/10 | 4 | 4 | 8 | 4 | 8 |
| 085 | 8 | 2 | 9 | 12 | 2 | 9 |
| 086 | 9/10 | 8 | 4 | 12 | 4 | 2 |
| 087 | 8 | 2 | 12 | 12 | 9 | 12 |
| 088 | 9/10 | 4 | 16 | 8 | 9 | 9 |
| 089 | 8 | 0 | 8 | 8 | 9 | 8 |
| 090 | 9/10 | 4 | 12 | 9 | 6 | 6 |
| 091 | 9/10 | 4 | 12 | 12 | 4 | 8 |
| 092 | 9/10 | 2 | 8 | 8 | 2 | 4 |
| 093 | 9/10 | 8 | 4 | 12 | 2 | 9 |
| 094 | 9/10 | 4 | 12 | 9 | 4 | 9 |
| 095 | 9/10 | 4 | 4 | 3 | 4 | 12 |
| 096 | 9/10 | 4 | 12 | 8 | 8 | 6 |
| 097 | 9/10 | 4 | 8 | 3 | 2 | 4 |
| 098 | 9/10 | 12 | 6 | 0 |  |  |
| 099 | 8 | 8 | 9 | 12 | 4 | 6 |
| 100 | 7=3+4 | 12 | 6 | 12 | 2 | 6 |
| 101 | 8 | 6 |  | 0 |  | 6 |
| 102 | 9/10 | 4 | 12 | 0 |  | 9 |
| 103 | 8 | 12 | 4 | 8 |  | 12 |
| 104 | 9/10 | 6 | 4 | 8 | 4 | 6 |
| 105 | 9/10 | 8 | 3 | 8 | 4 | 12 |
| 106 | 8 | 8 | 6 | 12 | 6 | 12 |
| 107 | 8 | 8 | 3 | 9 | 2 | 9 |
| 108 | 9/10 | 12 | 4 | 6 | 4 | 3 |
| 109 | 9/10 | 9 | 6 | 12 | 2 | 6 |
| 110 | 9/10 | 8 | 12 | 8 | 3 | 6 |
| 111 | 8 | 4 | 8 | 12 | 6 | 12 |
| 112 | 9/10 | 8 | 12 | 9 | 4 | 9 |
| 113 | 9/10 | 12 | 12 | 12 | 4 | 12 |
| 114 | 8 | 12 | 6 | 0 | 6 | 12 |
| 115 | 9/10 | 8 | 4 | 8 | 4 | 12 |
| 116 | 9/10 | 0 | 6 | 0 |  | 8 |
| 117 | 9/10 | 8 | 3 | 8 | 4 | 9 |
| 118 | 8 | 8 | 4 | 8 | 6 | 8 |
| 119 | 6 | 12 | 3 | 6 | 2 | 6 |
| 120 | 7=3+4 | 3 | 6 | 12 | 4 | 9 |
| 121 | 7=3+4 | 12 | 9 | 12 | 9 | 12 |
| 122 | 7=3+4 | 6 | 6 | 8 | 2 | 3 |
| 123 | 7=3+4 | 9 | 4 | 6 | 4 | 12 |
| 124 | 7=3+4 | 6 | 2 | 4 | 4 | 6 |
| 125 | 7=3+4 | 12 | 4 | 9 | 8 | 8 |
| 126 | 6 | 4 | 1 | 2 | 0 | 4 |
| 127 | 6 | 6 | 0 | 2 | 0 | 8 |
| 128 | 9/10 | 6 | 6 | 12 | 4 | 12 |
| 129 | 7=4+3 | 12 | 8 | 8 | 3 | 8 |
| 130 | 9/10 | 12 | 12 | 2 | 3 | 12 |
| 131 | 9/10 | 12 | 12 | 9 | 6 | 9 |
| 132 | 9/10 | 4 | 8 | 4 | 4 | 9 |
| 133 | 7=3+4 | 6 | 9 | 3 | 4 | 8 |
| 134 | 7=3+4 | 4 | 2 | 4 | 4 | 8 |
| 135 | 7=4+3 | 3 | 1 | 3 | 4 | 6 |
| 136 | 7=3+4 | 4 | 3 | 4 | 0 | 6 |
| 137 | 6 | 12 | 8 | 12 | 6 | 12 |

1. Raw data used to determine the difference in biomarker score between tumour tissue and corresponding “healthy” tissue taken from the same patient (Figure 2).

| Patient Number | Normal/Cancer | STEAP1 Score | STEAP2 Score | STEAP3 Score | STEAP4 Score | DMT1 Score |
| --- | --- | --- | --- | --- | --- | --- |
| 001 | Cancer | 4 | 4 | 8 | 0 | 6 |
| 002 | Cancer | 4 | 0 | 4 | 4 | 4 |
| 003 | Cancer | 4 | 4 | 8 | 12 | 12 |
| 004 | Cancer | 12 | 4 | 8 | 4 | 9 |
| 005 | Cancer | 0 | 6 | 8 | 4 | 8 |
| 006 | Cancer | 6 | 2 | 8 | 2 | 12 |
| 007 | Cancer | 8 | 4 | 8 | 0 | 12 |
| 008 | Cancer | 4 | 9 | 12 | 4 | 6 |
| 009 | Cancer | 4 | 2 | 2 | 6 | 8 |
| 010 | Cancer | 2 | 2 | 4 | 4 | 9 |
| 011 | Cancer | 6 | 3 | 6 | 4 | 12 |
| 012 | Cancer | 0 | 4 | 4 | 3 | 4 |
| 013 | Cancer | 3 | 9 | 12 | 4 | 12 |
| 014 | Cancer | 4 | 8 | 12 | 4 | 9 |
| 015 | Cancer | 3 | 6 | 6 | 4 | 12 |
| 016 | Cancer | 4 | 4 | 2 | 4 | 9 |
| 017 | Cancer | 6 | 2 | 8 | 4 | 12 |
| 018 | Cancer | 6 | 6 | 6 | 4 | 12 |
| 019 | Cancer | 6 | 2 | 8 | 4 | 9 |
| 020 | Cancer | 2 | 4 | 8 | 4 | 8 |
| 021 | Cancer | 4 | 2 | 12 | 2 | 12 |
| 022 | Cancer | 0 | 12 | 4 | 3 | 6 |
| 023 | Cancer | 4 | 4 | 8 | 4 | 9 |
| 024 | Cancer | 4 | 1 | 6 | 8 | 8 |
| 025 | Cancer | 3 | 6 | 8 | 4 | 9 |
| 026 | Cancer | 9 | 4 | 12 | 6 | 9 |
| 027 | Cancer | 0 | 6 | 8 | 4 | 12 |
| 028 | Cancer | 3 | 4 | 4 | 4 | 6 |
| 029 | Cancer | 6 | 4 | 12 | 3 | 12 |
| 030 | Cancer | 0 | 0 | 3 | 4 | 8 |
| 031 | Cancer | 8 | 4 | 6 | 4 | 8 |
| 032 | Cancer | 12 | 1 | 4 | 2 | 6 |
| 033 | Cancer | 12 | 2 | 12 | 4 | 6 |
| 034 | Cancer | 4 | 3 | 8 | 4 | 12 |
| 035 | Cancer | 4 | 3 | 8 | 6 | 9 |
| 036 | Cancer | 0 | 4 | 4 | 0 | 8 |
| 037 | Cancer | 8 | 4 | 6 | 4 | 8 |
| 038 | Cancer | 4 | 6 | 9 | 4 | 9 |
| 039 | Cancer | 6 | 4 | 9 | 2 | 9 |
| 040 | Cancer | 4 | 6 | 8 | 4 | 9 |
| 041 | Cancer | 8 | 0 | 4 | 4 | 6 |
| 042 | Cancer | 9 | 0 | 4 | 4 | 8 |
| 043 | Cancer | 2 | 4 | 4 | 6 | 2 |
| 044 | Cancer | 8 | 4 | 8 | 4 | 12 |
| 045 | Cancer | 3 | 2 | 6 | 3 | 6 |
| 046 | Cancer | 8 | 4 | 12 | 4 | 8 |
| 047 | Cancer | 12 | 4 | 8 | 4 | 8 |
| 048 | Cancer | 2 | 0 | 4 | 2 | 4 |
| 049 | Cancer | 4 | 4 | 12 | 6 | 12 |
| 050 | Cancer | 8 | 2 | 6 | 4 | 9 |
| 051 | Cancer | 4 | 8 | 8 | 4 | 9 |
| 052 | Cancer | 12 | 4 | 12 | 8 | 9 |
| 053 | Cancer | 6 | 4 | 12 | 4 | 12 |
| 054 | Cancer | 6 | 3 | 4 | 3 | 9 |
| 055 | Cancer | 12 | 4 | 6 | 4 | 8 |
| 056 | Cancer | 6 | 3 | 4 | 4 | 9 |
| 057 | Cancer | 12 | 4 | 12 | 8 | 9 |
| 058 | Cancer | 12 | 4 | 8 | 4 | 4 |
| 059 | Cancer | 8 | 4 | 9 | 3 | 12 |
| 060 | Cancer | 6 | 2 | 6 | 2 | 6 |
| 061 | Cancer | 4 | 1 | 8 | 12 | 8 |
| 062 | Cancer | 4 | 4 | 12 | 2 | 12 |
| 063 | Cancer | 8 | 6 | 12 | 3 | 12 |
| 064 | Cancer | 0 | 4 | 4 | 6 | 12 |
| 065 | Cancer | 4 | 6 | 12 | 12 | 6 |
| 066 | Cancer | 6 | 9 | 8 | 8 | 9 |
| 067 | Cancer | 0 | 6 | 4 | 4 | 8 |
| 068 | Cancer | 8 | 2 | 8 | 4 | 9 |
| 069 | Cancer | 3 | 9 | 9 | 4 | 12 |
| 070 | Cancer | 8 | 2 | 3 | 6 | 6 |
| 071 | Cancer | 9 | 4 | 9 | 4 | 6 |
| 072 | Cancer | 6 | 3 | 4 | 6 | 12 |
| 073 | Cancer | 6 | 4 | 6 | 2 | 12 |
| 074 | Cancer | 12 | 6 | 12 | 8 | 12 |
| 075 | Cancer | 4 | 4 | 12 | 12 | 8 |
| 076 | Cancer | 4 | 12 | 4 | 0 | 6 |
| 077 | Cancer | 4 | 12 | 9 | 6 | 6 |
| 078 | Cancer | 4 | 9 | 9 | 9 | 12 |
| 079 | Cancer | 4 | 6 | 6 | 6 | 8 |
| 080 | Cancer | 4 | 4 | 12 | 9 | 12 |
| 081 | Cancer | 4 | 8 | 12 | 4 | 6 |
| 082 | Cancer | 4 | 9 | 6 | 4 | 8 |
| 083 | Cancer | 0 | 9 | 2 | 6 | 8 |
| 084 | Cancer | 4 | 4 | 8 | 4 | 8 |
| 085 | Cancer | 2 | 9 | 12 | 2 | 9 |
| 086 | Cancer | 8 | 4 | 12 | 4 | 2 |
| 087 | Cancer | 2 | 12 | 12 | 9 | 12 |
| 088 | Cancer | 4 | 16 | 8 | 9 | 9 |
| 089 | Cancer | 0 | 8 | 8 | 9 | 8 |
| 090 | Cancer | 4 | 12 | 9 | 6 | 6 |
| 091 | Cancer | 4 | 12 | 12 | 4 | 8 |
| 092 | Cancer | 2 | 8 | 8 | 2 | 4 |
| 093 | Cancer | 8 | 4 | 12 | 2 | 9 |
| 094 | Cancer | 4 | 12 | 9 | 4 | 9 |
| 095 | Cancer | 4 | 4 | 3 | 4 | 12 |
| 096 | Cancer | 4 | 12 | 8 | 8 | 6 |
| 097 | Cancer | 4 | 8 | 3 | 2 | 4 |
| 099 | Cancer | 8 | 9 | 12 | 4 | 6 |
| 100 | Cancer | 12 | 6 | 12 | 2 | 6 |
| 103 | Cancer | 12 | 4 | 8 |  | 12 |
| 104 | Cancer | 6 | 4 | 8 | 4 | 6 |
| 105 | Cancer | 8 | 3 | 8 | 4 | 12 |
| 106 | Cancer | 8 | 6 | 12 | 6 | 12 |
| 107 | Cancer | 8 | 3 | 9 | 2 | 9 |
| 108 | Cancer | 12 | 4 | 6 | 4 | 3 |
| 109 | Cancer | 9 | 6 | 12 | 2 | 6 |
| 110 | Cancer | 8 | 12 | 8 | 3 | 6 |
| 111 | Cancer | 4 | 8 | 12 | 6 | 12 |
| 112 | Cancer | 8 | 12 | 9 | 4 | 9 |
| 113 | Cancer | 12 | 12 | 12 | 4 | 12 |
| 115 | Cancer | 8 | 4 | 8 | 4 | 12 |
| 117 | Cancer | 8 | 3 | 8 | 4 | 9 |
| 118 | Cancer | 8 | 4 | 8 | 6 | 8 |
| 119 | Cancer | 12 | 3 | 6 | 2 | 6 |
| 120 | Cancer | 3 | 6 | 12 | 4 | 9 |
| 121 | Cancer | 12 | 9 | 12 | 9 | 12 |
| 122 | Cancer | 6 | 6 | 8 | 2 | 3 |
| 123 | Cancer | 9 | 4 | 6 | 4 | 12 |
| 124 | Cancer | 6 | 2 | 4 | 4 | 6 |
| 125 | Cancer | 12 | 4 | 9 | 8 | 8 |
| 126 | Cancer | 4 | 1 | 2 | 0 | 4 |
| 127 | Cancer | 6 | 0 | 2 | 0 | 8 |
| 128 | Cancer | 6 | 6 | 12 | 4 | 12 |
| 129 | Cancer | 12 | 8 | 8 | 3 | 8 |
| 130 | Cancer | 12 | 12 | 2 | 3 | 12 |
| 131 | Cancer | 12 | 12 | 9 | 6 | 9 |
| 132 | Cancer | 4 | 8 | 4 | 4 | 9 |
| 133 | Cancer | 6 | 9 | 3 | 4 | 8 |
| 134 | Cancer | 4 | 2 | 4 | 4 | 8 |
| 135 | Cancer | 3 | 1 | 3 | 4 | 6 |
| 136 | Cancer | 4 | 3 | 4 | 0 | 6 |
| 137 | Cancer | 12 | 8 | 12 | 6 | 12 |
| 031 | Normal | 4 | 1 | 12 | 0 | 4 |
| 078 | Normal | 1 | 4 | 12 | 0 | 4 |
| 083 | Normal | 0 | 8 | 6 | 0 | 2 |
| 087 | Normal | 1 | 8 | 8 | 3 | 6 |
| 091 | Normal | 0 | 6 | 9 | 0 | 4 |
| 100 | Normal | 4 | 6 | 4 | 4 | 4 |
| 118 | Normal | 4 | 0 | 4 | 3 | 4 |
| 120 | Normal | 4 | 2 | 8 | 4 | 4 |
| 122 | Normal | 4 | 4 | 8 | 3 | 6 |
| 123 | Normal | 4 | 2 | 6 | 4 | 8 |
| 124 | Normal | 4 | 0 | 4 | 0 | 4 |
| 129 | Normal | 4 | 2 | 2 | 0 | 8 |
| 133 | Normal | 4 | 6 | 6 | 2 | 8 |
| 136 | Normal | 4 | 6 | 6 | 2 | 6 |
| 138 | Normal | 1 | 0 | 12 | 0 | 4 |
| 139 | Normal | 0 | 0 | 4 | 0 | 2 |
| 140 | Normal | 3 | 9 | 9 | 2 | 12 |
| 141 | Normal | 0 | 3 | 6 | 0 | 6 |
| 142 | Normal | 4 | 9 | 6 | 4 | 8 |
| 143 | Normal | 0 | 0 | 4 | 0 | 4 |
| 144 | Normal | 0 | 0 | 4 | 2 | 0 |
| 145 | Normal | 0 | 2 | 8 | 0 | 4 |
| 146 | Normal | 0 | 3 | 6 | 0 | 4 |
| 147 | Normal | 2 | 3 | 12 | 0 | 8 |
| 148 | Normal | 0 | 0 | 4 | 0 | 4 |
| 149 | Normal | 0 | 0 | 4 | 0 | 4 |
| 150 | Normal | 0 | 0 | 12 | 0 | 4 |
| 151 | Normal | 0 | 0 | 9 | 4 | 4 |
| 152 | Normal | 0 | 0 | 4 | 0 | 4 |
| 153 | Normal | 4 | 0 | 8 | 0 | 8 |
| 154 | Normal | 3 | 2 | 12 | 4 | 4 |
| 155 | Normal | 2 | 0 | 4 | 0 | 4 |
| 156 | Normal | 4 | 2 | 9 | 4 | 6 |

1. Raw data used to show correlations between biomarker score, PSA and age (Figure 3).

| Patient Number | STEAP1 Score | STEAP2 Score | STEAP3 Score | STEAP4 Score | DMT1 Score | PSA | LogPSA | Age |
| --- | --- | --- | --- | --- | --- | --- | --- | --- |
| 001 | 4 | 4 | 8 | 0 | 6 | 5 | 0.7 | 66 |
| 002 | 4 | 0 | 4 | 4 | 4 | 109.2 | 2.04 | 77 |
| 003 | 4 | 4 | 8 | 12 | 12 | 6.6 | 0.82 | 66 |
| 004 | 12 | 4 | 8 | 4 | 9 | 7.1 | 0.85 | 49 |
| 005 | 0 | 6 | 8 | 4 | 8 | 10.4 | 1.02 | 59 |
| 006 | 6 | 2 | 8 | 2 | 12 | 8.2 | 0.91 | 68 |
| 007 | 8 | 4 | 8 | 0 | 12 | 35.9 | 1.56 | 76 |
| 008 | 4 | 9 | 12 | 4 | 6 | 12 | 1.08 | 70 |
| 009 | 4 | 2 | 2 | 6 | 8 | 16 | 1.2 | 63 |
| 010 | 2 | 2 | 4 | 4 | 9 | 20 | 1.3 | 67 |
| 011 | 6 | 3 | 6 | 4 | 12 | 23.1 | 1.36 | 43 |
| 012 | 0 | 4 | 4 | 3 | 4 | 9.8 | 0.99 | 66 |
| 013 | 3 | 9 | 12 | 4 | 12 | 6.2 | 0.79 | 57 |
| 014 | 4 | 8 | 12 | 4 | 9 | 12.3 | 1.09 | 65 |
| 015 | 3 | 6 | 6 | 4 | 12 | 8.3 | 0.92 | 68 |
| 016 | 4 | 4 | 2 | 4 | 9 | 8.8 | 0.94 | 69 |
| 017 | 6 | 2 | 8 | 4 | 12 | 6.2 | 0.79 | 68 |
| 018 | 6 | 6 | 6 | 4 | 12 | 12 | 1.08 | 61 |
| 019 | 6 | 2 | 8 | 4 | 9 | 7.8 | 0.89 | 58 |
| 020 | 2 | 4 | 8 | 4 | 8 | 7.9 | 0.9 | 67 |
| 021 | 4 | 2 | 12 | 2 | 12 | 14 | 1.15 | 72 |
| 022 | 0 | 12 | 4 | 3 | 6 | 4.5 | 0.65 | 51 |
| 023 | 4 | 4 | 8 | 4 | 9 | 2.2 | 0.34 | 58 |
| 024 | 4 | 1 | 6 | 8 | 8 | 5.5 | 0.74 | 68 |
| 025 | 3 | 6 | 8 | 4 | 9 | 26.8 | 1.43 | 53 |
| 026 | 3 | 4 | 4 | 4 | 6 | 9.1 | 0.96 | 57 |
| 027 | 6 | 4 | 12 | 3 | 12 | 6.2 | 0.79 | 65 |
| 028 | 0 | 0 | 3 | 4 | 8 | 14.1 | 1.15 | 68 |
| 029 | 8 | 4 | 6 | 4 | 8 | 11.3 | 1.05 | 74 |
| 030 | 12 | 1 | 4 | 2 | 6 | 2.8 | 0.45 | 60 |
| 031 | 12 | 2 | 12 | 4 | 6 | 3.1 | 0.49 | 56 |
| 032 | 4 | 3 | 8 | 4 | 12 | 23 | 1.36 | 48 |
| 033 | 4 | 3 | 8 | 6 | 9 | 9 | 0.95 | 59 |
| 034 | 0 | 4 | 4 | 0 | 8 | 6.7 | 0.83 | 65 |
| 035 | 8 | 4 | 6 | 4 | 8 | 12.7 | 1.1 | 66 |
| 036 | 4 | 6 | 9 | 4 | 9 | 17.1 | 1.23 | 71 |
| 037 | 6 | 4 | 9 | 2 | 9 | 7.5 | 0.88 | 69 |
| 038 | 4 | 6 | 8 | 4 | 9 | 4.7 | 0.67 | 67 |
| 039 | 8 | 0 | 4 | 4 | 6 | 8.3 | 0.92 | 60 |
| 040 | 9 | 0 | 4 | 4 | 8 | 3 | 0.48 | 59 |
| 042 | 2 | 4 | 4 | 6 | 2 | 8 | 0.9 | 64 |
| 043 | 8 | 4 | 8 | 4 | 12 | 7 | 0.85 | 54 |
| 044 | 3 | 2 | 6 | 3 | 6 | 12.7 | 1.1 | 84 |
| 045 | 8 | 4 | 12 | 4 | 8 | 3.2 | 0.51 | 49 |
| 046 | 12 | 4 | 8 | 4 | 8 | 2.2 | 0.34 | 63 |
| 047 | 2 | 0 | 4 | 2 | 4 | 8.9 | 0.95 | 75 |
| 048 | 4 | 4 | 12 | 6 | 12 | 11 | 1.04 | 64 |
| 049 | 8 | 2 | 6 | 4 | 9 | 12 | 1.08 | 64 |
| 050 | 4 | 8 | 8 | 4 | 9 | 11 | 1.04 | 67 |
| 051 | 12 | 4 | 12 | 8 | 9 | 5.9 | 0.77 | 50 |
| 052 | 6 | 4 | 12 | 4 | 12 | 11 | 1.04 | 72 |
| 053 | 6 | 3 | 4 | 3 | 9 | 3.8 | 0.58 | 57 |
| 054 | 12 | 4 | 6 | 4 | 8 | 3.5 | 0.54 | 65 |
| 055 | 6 | 3 | 4 | 4 | 9 | 6.9 | 0.84 | 56 |
| 056 | 12 | 4 | 12 | 8 | 9 | 5.6 | 0.75 | 69 |
| 057 | 12 | 4 | 8 | 4 | 4 | 11 | 1.04 | 49 |
| 058 | 8 | 4 | 9 | 3 | 12 | 7.2 | 0.86 | 64 |
| 059 | 6 | 2 | 6 | 2 | 6 | 6.3 | 0.8 | 49 |
| 060 | 4 | 1 | 8 | 12 | 8 | 7.7 | 0.89 | 68 |
| 061 | 4 | 4 | 12 | 2 | 12 | 14.7 | 1.17 | 67 |
| 062 | 8 | 6 | 12 | 3 | 12 | 5.9 | 0.77 | 63 |
| 063 | 0 | 4 | 4 | 6 | 12 | 4.5 | 0.65 | 64 |
| 064 | 4 | 6 | 12 | 12 | 6 | 6.4 | 0.81 | 65 |
| 065 | 6 | 9 | 8 | 8 | 9 | 6.2 | 0.79 | 58 |
| 066 | 0 | 6 | 4 | 4 | 8 | 7.8 | 0.89 | 64 |
| 067 | 8 | 2 | 8 | 4 | 9 | 15.1 | 1.18 | 64 |
| 068 | 3 | 9 | 9 | 4 | 12 | 22.5 | 1.35 | 59 |
| 069 | 8 | 2 | 3 | 6 | 6 | 8 | 0.9 | 56 |
| 070 | 9 | 4 | 9 | 4 | 6 | 13.4 | 1.13 | 71 |
| 071 | 6 | 3 | 4 | 6 | 12 | 29 | 1.46 | 55 |
| 072 | 6 | 4 | 6 | 2 | 12 | 15.7 | 1.2 | 64 |
| 073 | 12 | 6 | 12 | 8 | 12 | 22 | 1.34 | 59 |
| 074 | 6 | 8 | 12 | 4 | 12 | 5.7 | 0.76 | 58 |
| 075 | 4 | 4 | 4 | 12 | 8 | 9.5 | 0.98 | 63 |
| 076 | 4 | 12 | 4 | 0 | 6 | 11 | 1.04 | 66 |
| 077 | 4 | 12 | 9 | 6 | 6 | 19.5 | 1.29 | 71 |
| 078 | 4 | 9 | 9 | 9 | 12 | 4.3 | 0.63 | 73 |
| 079 | 4 | 6 | 6 | 6 | 8 | 16.4 | 1.21 | 71 |
| 080 | 4 | 4 | 12 | 9 | 12 | 10.3 | 1.01 | 69 |
| 081 | 4 | 8 | 12 | 4 | 6 | 26.3 | 1.42 | 52 |
| 082 | 4 | 9 | 6 | 4 | 8 | 13.9 | 1.14 | 68 |
| 083 | 0 | 9 | 2 | 6 | 8 | 19.4 | 1.29 | 51 |
| 084 | 4 | 4 | 8 | 4 | 8 | 5.6 | 0.75 | 61 |
| 085 | 2 | 9 | 12 | 2 | 9 | 17.7 | 1.25 | 65 |
| 086 | 8 | 4 | 12 | 4 | 2 | 4.5 | 0.65 | 65 |
| 087 | 2 | 12 | 12 | 9 | 12 | 6.9 | 0.84 | 52 |
| 088 | 4 | 16 | 8 | 9 | 9 | 2.2 | 0.34 | 47 |
| 089 | 0 | 8 | 8 | 9 | 8 | 6.9 | 0.84 | 64 |
| 090 | 4 | 12 | 9 | 6 | 6 | 12.8 | 1.11 | 61 |
| 091 | 4 | 12 | 12 | 4 | 8 | 8.9 | 0.95 | 68 |
| 092 | 2 | 8 | 8 | 2 | 4 | 18.5 | 1.27 | 58 |
| 093 | 8 | 4 | 12 | 2 | 9 | 5.2 | 0.72 | 70 |
| 094 | 4 | 12 | 9 | 4 | 9 | 13.8 | 1.14 | 65 |
| 095 | 4 | 4 | 3 | 4 | 12 | 10.1 | 1 | 79 |
| 096 | 4 | 12 | 8 | 8 | 6 | 12.2 | 1.09 | 65 |
| 097 | 4 | 8 | 3 | 2 | 4 | 7.2 | 0.86 | 64 |
| 099 | 8 | 9 | 12 | 4 | 6 | 24 | 1.38 | 61 |
| 100 | 12 | 6 | 12 | 2 | 6 | 0 | 0 | 57 |
| 104 | 6 | 4 | 8 | 4 | 6 | 14.7 | 1.17 | 61 |
| 105 | 8 | 3 | 8 | 4 | 12 | 8 | 0.9 | 57 |
| 106 | 8 | 6 | 12 | 6 | 12 | 7.4 | 0.87 | 57 |
| 107 | 8 | 3 | 9 | 2 | 9 | 6 | 0.78 | 61 |
| 108 | 12 | 4 | 6 | 4 | 3 | 14 | 1.15 | 59 |
| 109 | 9 | 6 | 12 | 2 | 6 | 6.3 | 0.8 | 66 |
| 110 | 8 | 12 | 8 | 3 | 6 | 9 | 0.95 | 68 |
| 111 | 4 | 8 | 12 | 6 | 12 | 11.2 | 1.05 | 68 |
| 112 | 8 | 12 | 9 | 4 | 9 | 12 | 1.08 | 62 |
| 113 | 12 | 12 | 12 | 4 | 12 | 8.7 | 0.94 | 65 |
| 115 | 8 | 4 | 8 | 4 | 12 | 9.3 | 0.97 | 66 |
| 118 | 8 | 4 | 8 | 6 | 8 | 8.5 | 0.93 | 61 |
| 119 | 12 | 3 | 6 | 2 | 6 | 0 | 0 | 67 |
| 120 | 3 | 6 | 12 | 4 | 9 | 0 | 0 | 61 |
| 121 | 12 | 9 | 12 | 9 | 12 | 28.8 | 1.46 | 66 |
| 122 | 6 | 6 | 8 | 2 | 3 | 5.6 | 0.75 | 54 |
| 123 | 9 | 4 | 6 | 4 | 12 | 4.4 | 0.64 | 58 |
| 124 | 6 | 2 | 4 | 4 | 6 | 0 | 0 | 56 |
| 125 | 12 | 4 | 9 | 8 | 8 | 29.2 | 1.47 | 54 |
| 126 | 4 | 1 | 2 | 0 | 4 | 1.9 | 0.28 | 58 |
| 127 | 6 | 0 | 2 | 0 | 8 | 7.7 | 0.89 | 58 |
| 128 | 6 | 6 | 12 | 4 | 12 | 12.1 | 1.08 | 78 |
| 129 | 12 | 8 | 8 | 3 | 8 | 5.6 | 0.75 | 67 |
| 130 | 12 | 12 | 2 | 3 | 12 | 66.4 | 1.82 | 82 |
| 131 | 12 | 12 | 9 | 6 | 9 | 17.7 | 1.25 | 85 |
| 132 | 4 | 8 | 4 | 4 | 9 | 2.6 | 0.41 | 63 |
| 133 | 6 | 9 | 3 | 4 | 8 | 27.9 | 1.45 | 54 |
| 134 | 4 | 2 | 4 | 4 | 8 | 9.2 | 0.96 | 67 |
| 135 | 3 | 1 | 3 | 4 | 6 | 9.7 | 0.99 | 69 |
| 136 | 4 | 3 | 4 | 0 | 6 | 15.5 | 1.19 | 70 |
| 137 | 12 | 8 | 12 | 6 | 12 | 3.6 | 0.56 | 62 |
| 157 | 0 | 6 | 8 | 4 | 12 | 8.6 | 0.93 | 61 |

1. Raw data used to show the effect of biomarker score on the chance of relapse. Scores split into groups: Low (0-2), Medium (3-6) and High (8-12) (Figure 4).

| Patient Number | Relapse? | STEAP1 Score | STEAP2 Score | STEAP3 Score | STEAP4 Score | DMT1 Score | Gleason | PSA | LogPSA | Age |
| --- | --- | --- | --- | --- | --- | --- | --- | --- | --- | --- |
| 001 | Yes | Medium | Medium | High | Low | Medium | 7=3+4 | 5 | 0.7 | 66 |
| 002 | No | Medium | Low | Medium | Medium | Medium | 9/10 | 109.2 | 2.04 | 77 |
| 003 | No | Medium | Medium | High | High | High | 7=4+3 | 6.6 | 0.82 | 66 |
| 004 | No | High | Medium | High | Medium | High | 6 | 7.1 | 0.85 | 49 |
| 005 | No | Low | Medium | High | Medium | High | 8 | 10.4 | 1.02 | 59 |
| 006 | No | Medium | Low | High | Low | High | 8 | 8.2 | 0.91 | 68 |
| 007 | Yes | High | Medium | High | Low | High | 9/10 | 35.9 | 1.56 | 76 |
| 008 | No | Medium | High | High | Medium | Medium | 6 | 12 | 1.08 | 70 |
| 009 | No | Medium | Low | Low | Medium | High | 7=4+3 | 16 | 1.2 | 63 |
| 010 | No | Low | Low | Medium | Medium | High | 9/10 | 20 | 1.3 | 67 |
| 011 | Yes | Medium | Medium | Medium | Medium | High | 9/10 | 23.1 | 1.36 | 43 |
| 012 | No | Low | Medium | Medium | Medium | Medium | 6 | 9.8 | 0.99 | 66 |
| 013 | No | Medium | High | High | Medium | High | 7=4+3 | 6.2 | 0.79 | 57 |
| 014 | No | Medium | High | High | Medium | High | 9/10 | 12.3 | 1.09 | 65 |
| 015 | No | Medium | Medium | Medium | Medium | High | 6 | 8.3 | 0.92 | 68 |
| 016 | No | Medium | Medium | Low | Medium | High | 7=3+4 | 8.8 | 0.94 | 69 |
| 017 | No | Medium | Low | High | Medium | High | 9/10 | 6.2 | 0.79 | 68 |
| 018 | No | Medium | Medium | Medium | Medium | High | 7=3+4 | 12 | 1.08 | 61 |
| 019 | No | Medium | Low | High | Medium | High | 8 | 7.8 | 0.89 | 58 |
| 020 | No | Low | Medium | High | Medium | High | 9/10 | 7.9 | 0.9 | 67 |
| 021 | Yes | Medium | Low | High | Low | High | 8 | 14 | 1.15 | 72 |
| 022 | Yes | Low | High | Medium | Medium | Medium | 9/10 | 4.5 | 0.65 | 51 |
| 023 | No | Medium | Medium | High | Medium | High | 6 | 2.2 | 0.34 | 58 |
| 024 | No | Medium | Low | Medium | High | High | 7=3+4 | 5.5 | 0.74 | 68 |
| 025 | No | Medium | Medium | High | Medium | High | 8 | 26.8 | 1.43 | 53 |
| 027 | Yes | Low | Medium | High | Medium | High | 8 | 8.6 | 0.93 | 61 |
| 028 | No | Medium | Medium | Medium | Medium | Medium | 9/10 | 9.1 | 0.96 | 57 |
| 029 | No | Medium | Medium | High | Medium | High | 7=3+4 | 6.2 | 0.79 | 65 |
| 030 | No | Low | Low | Medium | Medium | High | 7=3+4 | 14.1 | 1.15 | 68 |
| 031 | No | High | Medium | Medium | Medium | High | 8 | 11.3 | 1.05 | 74 |
| 032 | Yes | High | Low | Medium | Low | Medium | 6 | 2.8 | 0.45 | 60 |
| 033 | No | High | Low | High | Medium | Medium | 8 | 3.1 | 0.49 | 56 |
| 034 | No | Medium | Medium | High | Medium | High | 7=3+4 | 23 | 1.36 | 48 |
| 035 | No | Medium | Medium | High | Medium | High | 9/10 | 9 | 0.95 | 59 |
| 036 | Yes | Low | Medium | Medium | Low | High | 8 | 6.7 | 0.83 | 65 |
| 037 | Yes | High | Medium | Medium | Medium | High | 6 | 12.7 | 1.1 | 66 |
| 038 | No | Medium | Medium | High | Medium | High | 7=3+4 | 17.1 | 1.23 | 71 |
| 039 | No | Medium | Medium | High | Low | High | 6 | 7.5 | 0.88 | 69 |
| 040 | No | Medium | Medium | High | Medium | High | 7=3+4 | 4.7 | 0.67 | 67 |
| 041 | Yes | High | Low | Medium | Medium | High | 7=4+3 | 8.3 | 0.92 | 60 |
| 042 | No | High | Low | Medium | Medium | High | 9/10 | 3 | 0.48 | 59 |
| 043 | No | Low | Medium | Medium | Medium |  | 6 | 8 | 0.9 | 64 |
| 044 | No | High | Medium | High | Medium | High | 7=4+3 | 7 | 0.85 | 54 |
| 045 | No | Medium | Low | Medium | Medium | Medium | 9/10 | 12.7 | 1.1 | 84 |
| 046 | Yes | High | Medium | High | Medium | High | 6 | 3.2 | 0.51 | 49 |
| 047 | No | High | Medium | High | Medium | High | 7=4+3 | 2.2 | 0.34 | 63 |
| 048 | Yes | Low | Low | Medium | Low | Medium | 7=4+3 | 8.9 | 0.95 | 75 |
| 049 | No | Medium | Medium | High | Medium | High | 9/10 | 11 | 1.04 | 64 |
| 050 | No | High | Low | Medium | Medium | High | 6 | 12 | 1.08 | 64 |
| 051 | No | Medium | High | High | Medium | High | 7=3+4 | 11 | 1.04 | 67 |
| 052 | No | High | Medium | High | High | High | 7=3+4 | 5.9 | 0.77 | 50 |
| 053 | No | Medium | Medium | High | Medium | High | 7=3+4 | 11 | 1.04 | 72 |
| 054 | No | Medium | Medium | Medium | Medium | High | 6 | 3.8 | 0.58 | 57 |
| 055 | No | High | Medium | Medium | Medium | High | 7=4+3 | 3.5 | 0.54 | 65 |
| 056 | No | Medium | Medium | Medium | Medium | High | 7=3+4 | 6.9 | 0.84 | 56 |
| 057 | No | High | Medium | High | High | High | 6 | 5.6 | 0.75 | 69 |
| 058 | No | High | Medium | High | Medium | Medium | 7=3+4 | 11 | 1.04 | 49 |
| 059 | No | High | Medium | High | Medium | High | 7=3+4 | 7.2 | 0.86 | 64 |
| 060 | No | Medium | Low | Medium | Low | Medium | 6 | 6.3 | 0.8 | 49 |
| 061 | No | Medium | Low | High | High | High | 7=4+3 | 7.7 | 0.89 | 68 |
| 062 | No | Medium | Medium | High | Low | High | 7=3+4 | 14.7 | 1.17 | 67 |
| 063 | No | High | Medium | High | Medium | High | 6 | 5.9 | 0.77 | 63 |
| 064 | No | Low | Medium | Medium | Medium | High | 7=3+4 | 4.5 | 0.65 | 64 |
| 065 | No | Medium | Medium | High | High | Medium | 8 | 6.4 | 0.81 | 65 |
| 066 | No | Medium | High | High | High | High | 7=3+4 | 6.2 | 0.79 | 58 |
| 067 | No | Low | Medium | Medium | Medium | High | 7=3+4 | 7.8 | 0.89 | 64 |
| 068 | No | High | Low | High | Medium | High | 7=3+4 | 15.1 | 1.18 | 64 |
| 069 | No | Medium | High | High | Medium | High | 7=3+4 | 22.5 | 1.35 | 59 |
| 070 | No | High | Low | Medium | Medium | Medium | 6 | 8 | 0.9 | 56 |
| 071 | No | High | Medium | High | Medium | Medium | 7=3+4 | 13.4 | 1.13 | 71 |
| 072 | No | Medium | Medium | Medium | Medium | High | 7=3+4 | 29 | 1.46 | 55 |
| 073 | No | Medium | Medium | Medium | Low | High | 7=3+4 | 15.7 | 1.2 | 64 |
| 074 | Yes | High | Medium | High | High | High | 7=3+4 | 22 | 1.34 | 59 |
| 075 | Yes | Medium | Medium | Medium | High | High | 8 | 9.5 | 0.98 | 63 |
| 076 | No | Medium | High | Medium | Low | Medium | 8 | 11 | 1.04 | 66 |
| 077 | No | Medium | High | High | Medium | Medium | 8 | 19.5 | 1.29 | 71 |
| 078 | No | Medium | High | High | High | High | 9/10 | 4.3 | 0.63 | 73 |
| 079 | Yes | Medium | Medium | Medium | Medium | High | 9/10 | 16.4 | 1.21 | 71 |
| 080 | No | Medium | Medium | High | High | High | 8 | 10.3 | 1.01 | 69 |
| 081 | No | Medium | High | High | Medium | Medium | 9/10 | 26.3 | 1.42 | 52 |
| 082 | No | Medium | High | Medium | Medium | High | 8 | 13.9 | 1.14 | 68 |
| 083 | No | Low | High | Low | Medium | High | 9/10 | 19.4 | 1.29 | 51 |
| 084 | No | Medium | Medium | High | Medium | High | 9/10 | 5.6 | 0.75 | 61 |
| 085 | Yes | Low | High | High | Low | High | 8 | 17.7 | 1.25 | 65 |
| 086 | No | High | Medium | High | Medium | High | 9/10 | 4.5 | 0.65 | 65 |
| 087 | No | Low | High | High | High | High | 8 | 6.9 | 0.84 | 52 |
| 088 | No | Medium | High | High | High | High | 9/10 | 2.2 | 0.34 | 47 |
| 089 | No | Low | High | High | High | High | 8 | 6.9 | 0.84 | 64 |
| 090 | No | Medium | High | High | Medium | Medium | 9/10 | 12.8 | 1.11 | 61 |
| 091 | No | Medium | High | High | Medium | High | 9/10 | 8.9 | 0.95 | 68 |
| 092 | No | Low | High | High | Low | Medium | 9/10 | 18.5 | 1.27 | 58 |
| 093 | No | High | Medium | High | Low | High | 9/10 | 5.2 | 0.72 | 70 |
| 094 | No | Medium | High | High | Medium | High | 9/10 | 13.8 | 1.14 | 65 |
| 095 | Yes | Medium | Medium | Medium | Medium | High | 9/10 | 10.1 | 1 | 79 |
| 096 | No | Medium | High | High | High | Medium | 9/10 | 12.2 | 1.09 | 65 |
| 097 | No | Medium | High | Medium | Low | Medium | 9/10 | 7.2 | 0.86 | 64 |
| 099 | No | High | High | High | Medium | Medium | 8 | 24 | 1.38 | 61 |
| 100 | No | High | Medium | High | Low | Medium | 7=3+4 | 0 | 0 | 57 |
| 104 | No | Medium | Medium | High | Medium | Medium | 9/10 | 14.7 | 1.17 | 61 |
| 105 | Yes | High | Medium | High | Medium | High | 9/10 | 8 | 0.9 | 57 |
| 106 | Yes | High | Medium | High | Medium | High | 8 | 7.4 | 0.87 | 57 |
| 107 | Yes | High | Medium | High | Low | High | 8 | 6 | 0.78 | 61 |
| 108 | No | High | Medium | Medium | Medium | Medium | 9/10 | 14 | 1.15 | 59 |
| 109 | No | High | Medium | High | Low | Medium | 9/10 | 6.3 | 0.8 | 66 |
| 110 | No | High | High | High | Medium | Medium | 9/10 | 9 | 0.95 | 68 |
| 111 | Yes | Medium | High | High | Medium | High | 8 | 11.2 | 1.05 | 68 |
| 112 | No | High | High | High | Medium | High | 9/10 | 12 | 1.08 | 62 |
| 113 | No | High | High | High | Medium | High | 9/10 | 8.7 | 0.94 | 65 |
| 115 | No | High | Medium | High | Medium | High | 9/10 | 9.3 | 0.97 | 66 |
| 118 | No | High | Medium | High | Medium | High | 8 | 8.5 | 0.93 | 61 |
| 119 | No | High | Medium | Medium | Low | Medium | 6 | 0 | 0 | 67 |
| 120 | No | Medium | Medium | High | Medium | High | 7=3+4 | 0 | 0 | 61 |
| 121 | No | High | High | High | High | High | 7=3+4 | 28.8 | 1.46 | 66 |
| 122 | No | Medium | Medium | High | Low | Medium | 7=3+4 | 5.6 | 0.75 | 54 |
| 123 | No | High | Medium | Medium | Medium | High | 7=3+4 | 4.4 | 0.64 | 58 |
| 124 | No | Medium | Low | Medium | Medium | Medium | 7=3+4 | 0 | 0 | 56 |
| 125 | No | High | Medium | High | High | High | 7=3+4 | 29.2 | 1.47 | 54 |
| 126 | No | Medium | Low | Low | Low | High | 6 | 1.9 | 0.28 | 58 |
| 127 | No | Medium | Low | Low | Low | High | 6 | 7.7 | 0.89 | 58 |
| 128 | Yes | Medium | Medium | High | Medium | High | 9/10 | 12.1 | 1.08 | 78 |
| 129 | Yes | High | High | High | Medium | High | 7=4+3 | 5.6 | 0.75 | 67 |
| 130 | Yes | High | High | Low | Medium | High | 9/10 | 66.4 | 1.82 | 82 |
| 131 | Yes | High | High | High | Medium | High | 9/10 | 17.7 | 1.25 | 85 |
| 132 | Yes | Medium | High | Medium | Medium | High | 9/10 | 2.6 | 0.41 | 63 |
| 133 | No | Medium | High | Medium | Medium | High | 7=3+4 | 27.9 | 1.45 | 54 |
| 134 | No | Medium | Low | Medium | Medium | High | 7=3+4 | 9.2 | 0.96 | 67 |
| 135 | No | Medium | Low | Medium | Medium | Medium | 7=4+3 | 9.7 | 0.99 | 69 |
| 136 | No | Medium | Medium | Medium | Low | Medium | 7=3+4 | 15.5 | 1.19 | 70 |
| 137 | No | High | High | High | Medium | High | 6 | 3.6 | 0.56 | 62 |
| 158 | No | Medium | High | High | Medium | High | 7=3+4 | 5.7 | 0.76 | 58 |

1. Raw data used to carry out the binary logistic regression to determine the prognostic value of the biomarkers.

| Patient Number | Relapse? | STEAP1 Score | STEAP2 Score | STEAP3 Score | STEAP4 Score | DMT1 Score | PSA | LogPSA | Age | Gleason |
| --- | --- | --- | --- | --- | --- | --- | --- | --- | --- | --- |
| 001 | Yes | 4 | 4 | 8 | 0 | 6 | 5 | 0.7 | 66 | 7=3+4 |
| 007 | Yes | 8 | 4 | 8 | 0 | 12 | 35.9 | 1.56 | 76 | 9/10 |
| 011 | Yes | 6 | 3 | 6 | 4 | 12 | 23.1 | 1.36 | 43 | 9/10 |
| 021 | Yes | 4 | 2 | 12 | 2 | 12 | 14 | 1.15 | 72 | 8 |
| 022 | Yes | 0 | 12 | 4 | 3 | 6 | 4.5 | 0.65 | 51 | 9/10 |
| 027 | Yes | 0 | 6 | 8 | 4 | 12 | 8.6 | 0.93 | 61 | 8 |
| 032 | Yes | 12 | 1 | 4 | 2 | 6 | 2.8 | 0.45 | 60 | 6 |
| 036 | Yes | 0 | 4 | 4 | 0 | 8 | 6.7 | 0.83 | 65 | 8 |
| 037 | Yes | 8 | 4 | 6 | 4 | 8 | 12.7 | 1.1 | 66 | 6 |
| 041 | Yes | 8 | 0 | 4 | 4 | 12 | 8.3 | 0.92 | 60 | 7=4+3 |
| 046 | Yes | 8 | 4 | 12 | 4 | 12 | 3.2 | 0.51 | 49 | 6 |
| 048 | Yes | 2 | 0 | 4 | 2 | 12 | 8.9 | 0.95 | 75 | 7=4+3 |
| 074 | Yes | 12 | 6 | 12 | 8 | 6 | 22 | 1.34 | 59 | 7=3+4 |
| 075 | Yes | 4 | 4 | 4 | 12 | 12 | 9.5 | 0.98 | 63 | 8 |
| 079 | Yes | 4 | 6 | 6 | 6 | 6 | 16.4 | 1.21 | 71 | 9/10 |
| 085 | Yes | 2 | 9 | 12 | 2 | 8 | 17.7 | 1.25 | 65 | 8 |
| 095 | Yes | 4 | 4 | 3 | 4 | 8 | 10.1 | 1 | 79 | 9/10 |
| 105 | Yes | 8 | 3 | 8 | 4 | 12 | 8 | 0.9 | 57 | 9/10 |
| 106 | Yes | 8 | 6 | 12 | 6 | 12 | 7.4 | 0.87 | 57 | 8 |
| 107 | Yes | 8 | 3 | 9 | 2 | 9 | 6 | 0.78 | 61 | 8 |
| 111 | Yes | 4 | 8 | 12 | 6 | 12 | 11.2 | 1.05 | 68 | 8 |
| 128 | Yes | 6 | 6 | 12 | 4 | 12 | 12.1 | 1.08 | 78 | 9/10 |
| 129 | Yes | 12 | 8 | 8 | 3 | 8 | 5.6 | 0.75 | 67 | 4+3 |
| 130 | Yes | 12 | 12 | 2 | 3 | 12 | 66.4 | 1.82 | 82 | 9 |
| 131 | Yes | 12 | 12 | 9 | 6 | 9 | 17.7 | 1.25 | 85 | 9/10 |
| 132 | Yes | 4 | 8 | 4 | 4 | 9 | 2.6 | 0.41 | 63 | 9/10 |
| 002 | No | 4 | 0 | 4 | 4 | 4 | 109.2 | 2.04 | 77 | 9/10 |
| 003 | No | 4 | 4 | 8 | 12 | 12 | 6.6 | 0.82 | 66 | 7=4+3 |
| 004 | No | 12 | 4 | 8 | 4 | 9 | 7.1 | 0.85 | 49 | 6 |
| 005 | No | 0 | 6 | 8 | 4 | 8 | 10.4 | 1.02 | 59 | 8 |
| 006 | No | 6 | 2 | 8 | 2 | 12 | 8.2 | 0.91 | 68 | 8 |
| 008 | No | 4 | 9 | 12 | 4 | 6 | 12 | 1.08 | 70 | 6 |
| 009 | No | 4 | 2 | 2 | 6 | 8 | 16 | 1.2 | 63 | 7=4+3 |
| 010 | No | 2 | 2 | 4 | 4 | 9 | 20 | 1.3 | 67 | 9/10 |
| 012 | No | 0 | 4 | 4 | 3 | 4 | 9.8 | 0.99 | 66 | 6 |
| 013 | No | 3 | 9 | 12 | 4 | 12 | 6.2 | 0.79 | 57 | 7=4+3 |
| 014 | No | 4 | 8 | 12 | 4 | 9 | 12.3 | 1.09 | 65 | 9/10 |
| 015 | No | 3 | 6 | 6 | 4 | 12 | 8.3 | 0.92 | 68 | 6 |
| 016 | No | 4 | 4 | 2 | 4 | 9 | 8.8 | 0.94 | 69 | 7=3+4 |
| 017 | No | 6 | 2 | 8 | 4 | 12 | 6.2 | 0.79 | 68 | 9/10 |
| 018 | No | 6 | 6 | 6 | 4 | 12 | 12 | 1.08 | 61 | 7=3+4 |
| 019 | No | 6 | 2 | 8 | 4 | 9 | 7.8 | 0.89 | 58 | 8 |
| 020 | No | 2 | 4 | 8 | 4 | 8 | 7.9 | 0.9 | 67 | 9/10 |
| 023 | No | 4 | 4 | 8 | 4 | 9 | 2.2 | 0.34 | 58 | 6 |
| 024 | No | 4 | 1 | 6 | 8 | 8 | 5.5 | 0.74 | 68 | 7=3+4 |
| 025 | No | 3 | 6 | 8 | 4 | 9 | 26.8 | 1.43 | 53 | 8 |
| 028 | No | 3 | 4 | 4 | 4 | 6 | 9.1 | 0.96 | 57 | 9/10 |
| 029 | No | 6 | 4 | 12 | 3 | 12 | 6.2 | 0.79 | 65 | 7=3+4 |
| 030 | No | 0 | 0 | 3 | 4 | 8 | 14.1 | 1.15 | 68 | 7=3+4 |
| 031 | No | 8 | 4 | 6 | 4 | 8 | 11.3 | 1.05 | 74 | 8 |
| 033 | No | 12 | 2 | 12 | 4 | 6 | 3.1 | 0.49 | 56 | 8 |
| 034 | No | 4 | 3 | 8 | 4 | 12 | 23 | 1.36 | 48 | 7=3+4 |
| 035 | No | 4 | 3 | 8 | 6 | 9 | 9 | 0.95 | 59 | 9/10 |
| 038 | No | 4 | 6 | 9 | 4 | 9 | 17.1 | 1.23 | 71 | 7=3+4 |
| 039 | No | 6 | 4 | 9 | 2 | 9 | 7.5 | 0.88 | 69 | 6 |
| 040 | No | 4 | 6 | 8 | 4 | 9 | 4.7 | 0.67 | 67 | 7=3+4 |
| 042 | No | 9 | 0 | 4 | 4 | 8 | 3 | 0.48 | 59 | 9/10 |
| 043 | No | 2 | 4 | 4 | 6 | 2 | 8 | 0.9 | 64 | 6 |
| 044 | No | 8 | 4 | 8 | 4 | 12 | 7 | 0.85 | 54 | 7=4+3 |
| 045 | No | 3 | 2 | 6 | 3 | 6 | 12.7 | 1.1 | 84 | 9/10 |
| 047 | No | 12 | 4 | 8 | 4 | 8 | 2.2 | 0.34 | 63 | 7=4+3 |
| 049 | No | 4 | 4 | 12 | 6 | 12 | 11 | 1.04 | 64 | 9/10 |
| 050 | No | 8 | 2 | 6 | 4 | 9 | 12 | 1.08 | 64 | 6 |
| 051 | No | 4 | 8 | 8 | 4 | 9 | 11 | 1.04 | 67 | 7=3+4 |
| 052 | No | 12 | 4 | 12 | 8 | 9 | 5.9 | 0.77 | 50 | 7=3+4 |
| 053 | No | 6 | 4 | 12 | 4 | 12 | 11 | 1.04 | 72 | 7=3+4 |
| 054 | No | 6 | 3 | 4 | 3 | 9 | 3.8 | 0.58 | 57 | 6 |
| 055 | No | 12 | 4 | 6 | 4 | 8 | 3.5 | 0.54 | 65 | 7=4+3 |
| 056 | No | 6 | 3 | 4 | 4 | 9 | 6.9 | 0.84 | 56 | 7=3+4 |
| 057 | No | 12 | 4 | 12 | 8 | 9 | 5.6 | 0.75 | 69 | 6 |
| 058 | No | 12 | 4 | 8 | 4 | 4 | 11 | 1.04 | 49 | 7=3+4 |
| 059 | No | 8 | 4 | 9 | 3 | 12 | 7.2 | 0.86 | 64 | 7=3+4 |
| 060 | No | 6 | 2 | 6 | 2 | 6 | 6.3 | 0.8 | 49 | 6 |
| 061 | No | 4 | 1 | 8 | 12 | 8 | 7.7 | 0.89 | 68 | 7=4+3 |
| 062 | No | 4 | 4 | 12 | 2 | 12 | 14.7 | 1.17 | 67 | 7=3+4 |
| 063 | No | 8 | 6 | 12 | 3 | 12 | 5.9 | 0.77 | 63 | 6 |
| 064 | No | 0 | 4 | 4 | 6 | 12 | 4.5 | 0.65 | 64 | 7=3+4 |
| 065 | No | 4 | 6 | 12 | 12 | 6 | 6.4 | 0.81 | 65 | 8 |
| 066 | No | 6 | 9 | 8 | 8 | 9 | 6.2 | 0.79 | 58 | 7=3+4 |
| 067 | No | 0 | 6 | 4 | 4 | 8 | 7.8 | 0.89 | 64 | 7=3+4 |
| 068 | No | 8 | 2 | 8 | 4 | 9 | 15.1 | 1.18 | 64 | 7=3+4 |
| 069 | No | 3 | 9 | 9 | 4 | 12 | 22.5 | 1.35 | 59 | 7=3+4 |
| 070 | No | 8 | 2 | 3 | 6 | 6 | 8 | 0.9 | 56 | 6 |
| 071 | No | 9 | 4 | 9 | 4 | 6 | 13.4 | 1.13 | 71 | 7=3+4 |
| 072 | No | 6 | 3 | 4 | 6 | 12 | 29 | 1.46 | 55 | 7=3+4 |
| 073 | No | 6 | 4 | 6 | 2 | 12 | 15.7 | 1.2 | 64 | 7=3+4 |
| 076 | No | 4 | 12 | 4 | 0 | 6 | 11 | 1.04 | 66 | 8 |
| 077 | No | 4 | 12 | 9 | 6 | 6 | 19.5 | 1.29 | 71 | 8 |
| 078 | No | 4 | 9 | 9 | 9 | 12 | 4.3 | 0.63 | 73 | 9/10 |
| 080 | No | 4 | 4 | 12 | 9 | 12 | 10.3 | 1.01 | 69 | 8 |
| 081 | No | 4 | 8 | 12 | 4 | 6 | 26.3 | 1.42 | 52 | 9/10 |
| 082 | No | 4 | 9 | 6 | 4 | 8 | 13.9 | 1.14 | 68 | 8 |
| 083 | No | 0 | 9 | 2 | 6 | 8 | 19.4 | 1.29 | 51 | 9/10 |
| 084 | No | 4 | 4 | 8 | 4 | 8 | 5.6 | 0.75 | 61 | 9/10 |
| 086 | No | 8 | 4 | 12 | 4 | 2 | 4.5 | 0.65 | 65 | 9/10 |
| 087 | No | 2 | 12 | 12 | 9 | 12 | 6.9 | 0.84 | 52 | 8 |
| 088 | No | 4 | 16 | 8 | 9 | 9 | 2.2 | 0.34 | 47 | 9/10 |
| 089 | No | 0 | 8 | 8 | 9 | 8 | 6.9 | 0.84 | 64 | 8 |
| 090 | No | 4 | 12 | 9 | 6 | 6 | 12.8 | 1.11 | 61 | 9/10 |
| 091 | No | 4 | 12 | 12 | 4 | 8 | 8.9 | 0.95 | 68 | 9/10 |
| 092 | No | 2 | 8 | 8 | 2 | 4 | 18.5 | 1.27 | 58 | 9/10 |
| 093 | No | 8 | 4 | 12 | 2 | 9 | 5.2 | 0.72 | 70 | 9/10 |
| 094 | No | 4 | 12 | 9 | 4 | 9 | 13.8 | 1.14 | 65 | 9/10 |
| 096 | No | 4 | 12 | 8 | 8 | 6 | 12.2 | 1.09 | 65 | 9/10 |
| 097 | No | 4 | 8 | 3 | 2 | 4 | 7.2 | 0.86 | 64 | 9/10 |
| 099 | No | 8 | 9 | 12 | 4 | 6 | 24 | 1.38 | 61 | 8 |
| 104 | No | 6 | 4 | 8 | 4 | 6 | 14.7 | 1.17 | 61 | 9/10 |
| 108 | No | 12 | 4 | 6 | 4 | 3 | 14 | 1.15 | 59 | 9/10 |
| 109 | No | 9 | 6 | 12 | 2 | 6 | 6.3 | 0.8 | 66 | 9/10 |
| 110 | No | 8 | 12 | 8 | 3 | 6 | 9 | 0.95 | 68 | 9/10 |
| 112 | No | 8 | 12 | 9 | 4 | 9 | 12 | 1.08 | 62 | 9/10 |
| 113 | No | 12 | 12 | 12 | 4 | 12 | 8.7 | 0.94 | 65 | 9/10 |
| 115 | No | 8 | 4 | 8 | 4 | 12 | 9.3 | 0.97 | 66 | 9/10 |
| 118 | No | 8 | 4 | 8 | 6 | 8 | 8.5 | 0.93 | 61 | 8 |
| 119 | No | 12 | 9 | 12 | 9 | 12 | 28.8 | 1.46 | 66 | 3+4 |
| 122 | No | 6 | 6 | 8 | 2 | 3 | 5.6 | 0.75 | 54 | 3+4 |
| 123 | No | 9 | 4 | 6 | 4 | 12 | 4.4 | 0.64 | 58 | 3+4 |
| 125 | No | 12 | 4 | 9 | 8 | 8 | 29.2 | 1.47 | 54 | 3+4 |
| 126 | No | 4 | 1 | 2 | 0 | 4 | 1.9 | 0.28 | 58 | 6 |
| 127 | No | 6 | 0 | 2 | 0 | 8 | 7.7 | 0.89 | 58 | 6 |
| 133 | No | 6 | 9 | 3 | 4 | 8 | 27.9 | 1.45 | 54 | 3+4 |
| 134 | No | 4 | 2 | 4 | 4 | 8 | 9.2 | 0.96 | 67 | 3+4 |
| 135 | No | 3 | 1 | 3 | 4 | 6 | 9.7 | 0.99 | 69 | 4+3 |
| 136 | No | 4 | 3 | 4 | 0 | 6 | 15.5 | 1.19 | 70 | 3+4 |
| 137 | No | 12 | 8 | 12 | 6 | 12 | 3.6 | 0.56 | 62 | 6 |
| 158 | No | 6 | 8 | 12 | 4 | 12 | 5.7 | 0.76 | 58 | 7=3+4 |

1. Raw data used to construct the Kaplan Meier plots in Figure 5.

| Gleason | | | | STEAP1 | | | |
| --- | --- | --- | --- | --- | --- | --- | --- |
| Patient Number | Months | Log(Months) | Gleason | Patient Number | Months | Log(Months) | STEAP1 Score |
| 001 | 56 | 4.03 | 7=3+4 | 001 | 56 | 4.03 | Medium |
| 007 | 14 | 2.64 | 9/10 | 007 | 14 | 2.64 | High |
| 011 | 30 | 3.4 | 9/10 | 011 | 30 | 3.4 | Medium |
| 021 | 6 | 1.79 | 8 | 021 | 6 | 1.79 | Medium |
| 022 | 19 | 2.94 | 9/10 | 022 | 19 | 2.94 | Low |
| 026 | 14 | 2.64 | 8 | 026 | 14 | 2.64 | High |
| 027 | 9 | 2.2 | 9/10 | 027 | 9 | 2.2 | Low |
| 032 | 36 | 3.58 | 8 | 032 | 36 | 3.58 | High |
| 034 | 11 | 2.4 | 9/10 | 034 | 11 | 2.4 | Medium |
| 036 | 39 | 3.66 | 6 | 036 | 39 | 3.66 | Low |
| 046 | 2 | 0.69 | 7=4+3 | 046 | 2 | 0.69 | High |
| 048 | 27 | 3.3 | 9/10 | 048 | 27 | 3.3 | Low |
| 074 | 6 | 1.79 | 7=3+4 | 074 | 6 | 1.79 | High |
| 075 | 4 | 1.39 | 8 | 075 | 4 | 1.39 | Medium |
| 079 | 6 | 1.79 | 9/10 | 079 | 6 | 1.79 | Medium |
| 085 | 28 | 3.33 | 8 | 085 | 28 | 3.33 | Low |
| 095 | 3 | 1.1 | 9/10 | 095 | 3 | 1.1 | Medium |
| 103 | 12 | 2.48 | 8 | 103 | 12 | 2.48 | High |
| 105 | 17 | 2.83 | 9/10 | 105 | 17 | 2.83 | High |
| 106 | 23 | 3.14 | 8 | 106 | 23 | 3.14 | High |
| 107 | 20 | 3 | 8 | 107 | 20 | 3 | High |
| 111 | 44 | 3.78 | 8 | 111 | 44 | 3.78 | Medium |
| 129 | 56 | 4.03 | 7=4+3 | 129 | 56 | 4.03 | High |
| 131 | 31 | 3.43 | 8 | 130 | 81 | 4.39 | High |
| 132 | 18 | 2.89 | 9/10 | 131 | 31 | 3.43 | High |
| 145 | 54 | 3.99 | 6 | 132 | 18 | 2.89 | High |
| 157 | 8 | 2.08 | 9/10 | 145 | 54 | 3.99 | Medium |
| 158 | 47 | 3.85 | 8 | 157 | 8 | 2.08 | Low |
| 159 | 9 | 2.2 | 8 | 161 | 14 | 2.64 | High |
| 160 | 22 | 3.09 | 9/10 | 162 | 44 | 3.78 | Low |
| 161 | 14 | 2.64 | 7=3+4 | 163 | 30 | 3.4 | High |
| 162 | 44 | 3.78 | 9/10 | 164 | 1 | 0 | Medium |
| 163 | 30 | 3.4 | 8 | 165 | 57 | 4.04 | High |
| 164 | 1 | 0 | 9/10 | 166 | 2 | 0.69 | High |
| 165 | 57 | 4.04 | 8 |  |  |  |  |
| 166 | 2 | 0.69 | 9/10 |  |  |  |  |

| STEAP2 | | | | STEAP3 | | | |
| --- | --- | --- | --- | --- | --- | --- | --- |
| Patient Number | Months | Log(Months) | STEAP2 Score | Patient Number | Months | Log(Months) | STEAP3 Score |
| 001 | 56 | 4.03 | Medium | 001 | 56 | 4.03 | High |
| 007 | 14 | 2.64 | Medium | 007 | 14 | 2.64 | High |
| 011 | 30 | 3.4 | Medium | 011 | 30 | 3.4 | Medium |
| 021 | 6 | 1.79 | Low | 021 | 6 | 1.79 | High |
| 022 | 19 | 2.94 | High | 022 | 19 | 2.94 | Medium |
| 026 | 14 | 2.64 | Medium | 026 | 14 | 2.64 | High |
| 027 | 9 | 2.2 | Medium | 027 | 9 | 2.2 | High |
| 032 | 36 | 3.58 | Low | 032 | 36 | 3.58 | Medium |
| 034 | 11 | 2.4 | Medium | 034 | 11 | 2.4 | High |
| 036 | 39 | 3.66 | Medium | 036 | 39 | 3.66 | Medium |
| 046 | 2 | 0.69 | Medium | 046 | 2 | 0.69 | High |
| 048 | 27 | 3.3 | Low | 048 | 27 | 3.3 | Medium |
| 074 | 6 | 1.79 | Medium | 074 | 6 | 1.79 | High |
| 075 | 4 | 1.39 | Medium | 075 | 4 | 1.39 | High |
| 079 | 6 | 1.79 | Medium | 079 | 6 | 1.79 | Medium |
| 085 | 28 | 3.33 | High | 085 | 28 | 3.33 | High |
| 095 | 3 | 1.1 | Medium | 103 | 12 | 2.48 | Medium |
| 103 | 12 | 2.48 | Low | 105 | 17 | 2.83 | High |
| 105 | 17 | 2.83 | Medium | 107 | 20 | 3 | High |
| 106 | 23 | 3.14 | Medium | 111 | 44 | 3.78 | High |
| 107 | 20 | 3 | Medium | 129 | 56 | 4.03 | High |
| 111 | 44 | 3.78 | High | 130 | 81 | 4.39 | Low |
| 129 | 56 | 4.03 | High | 131 | 31 | 3.43 | High |
| 130 | 81 | 4.39 | High | 132 | 18 | 2.89 | Medium |
| 131 | 31 | 3.43 | High | 157 | 8 | 2.08 | Medium |
| 132 | 18 | 2.89 | Low | 161 | 14 | 2.64 | Medium |
| 145 | 54 | 3.99 | Medium | 162 | 44 | 3.78 | Medium |
| 161 | 14 | 2.64 | Medium | 163 | 30 | 3.4 | High |
| 162 | 44 | 3.78 | Low | 164 | 1 | 0 | High |
| 163 | 30 | 3.4 | Medium | 165 | 57 | 4.04 | High |
| 164 | 1 | 0 | Medium |  |  |  |  |
| 165 | 57 | 4.04 | Medium |  |  |  |  |
| 166 | 2 | 0.69 | Medium |  |  |  |  |

| STEAP4 | | | | DMT1 | | | |
| --- | --- | --- | --- | --- | --- | --- | --- |
| Patient Number | Months | Log(Months) | STEAP4 Score | Patient Number | Months | Log(Months) | STEAP4 Score |
| 001 | 56 | 4.03 | Low | 001 | 56 | 4.03 | Medium |
| 007 | 14 | 2.64 | Low | 007 | 14 | 2.64 | High |
| 011 | 30 | 3.4 | Medium | 011 | 30 | 3.4 | High |
| 021 | 6 | 1.79 | Low | 021 | 6 | 1.79 | High |
| 022 | 19 | 2.94 | Medium | 022 | 19 | 2.94 | Medium |
| 026 | 14 | 2.64 | Medium | 026 | 14 | 2.64 | High |
| 027 | 9 | 2.2 | Medium | 027 | 9 | 2.2 | High |
| 032 | 36 | 3.58 | Low | 032 | 36 | 3.58 | Medium |
| 034 | 11 | 2.4 | Medium | 034 | 11 | 2.4 | High |
| 036 | 39 | 3.66 | Low | 036 | 39 | 3.66 | High |
| 046 | 2 | 0.69 | Medium | 046 | 2 | 0.69 | High |
| 048 | 27 | 3.3 | Low | 048 | 27 | 3.3 | Medium |
| 074 | 6 | 1.79 | Medium | 074 | 6 | 1.79 | High |
| 075 | 4 | 1.39 | High | 075 | 4 | 1.39 | High |
| 079 | 6 | 1.79 | High | 079 | 6 | 1.79 | High |
| 085 | 28 | 3.33 | Low | 085 | 28 | 3.33 | High |
| 095 | 3 | 1.1 | Medium | 095 | 3 | 1.1 | High |
| 103 | 12 | 2.48 | Low | 103 | 12 | 2.48 | Medium |
| 105 | 17 | 2.83 | Medium | 105 | 17 | 2.83 | High |
| 106 | 23 | 3.14 | Medium | 106 | 23 | 3.14 | High |
| 111 | 44 | 3.78 | Medium | 107 | 20 | 3 | High |
| 129 | 56 | 4.03 | Medium | 111 | 44 | 3.78 | High |
| 130 | 81 | 4.39 | Medium | 129 | 56 | 4.03 | High |
| 131 | 31 | 3.43 | Medium | 130 | 81 | 4.39 | High |
| 132 | 18 | 2.89 | Medium | 131 | 31 | 3.43 | High |
| 157 | 8 | 2.08 | Low | 132 | 18 | 2.89 | Medium |
| 161 | 14 | 2.64 | Medium | 145 | 54 | 3.99 | High |
| 163 | 30 | 3.4 | Medium | 157 | 8 | 2.08 | Medium |
| 164 | 1 | 0 | Medium | 158 | 47 | 3.85 | Medium |
| 165 | 57 | 4.04 | Low | 159 | 9 | 2.2 | High |
|  |  |  |  | 160 | 22 | 3.09 | High |
|  |  |  |  | 161 | 14 | 2.64 | High |
|  |  |  |  | 163 | 30 | 3.4 | High |
|  |  |  |  | 164 | 1 | 0 | High |
|  |  |  |  | 165 | 57 | 4.04 | High |
